# Cost-benefit trade-offs in decision-making and learning

**DOI:** 10.1371/journal.pcbi.1007326

**Published:** 2019-09-06

**Authors:** Nura Sidarus, Stefano Palminteri, Valérian Chambon

**Affiliations:** 1 Institut Jean Nicod, Département d’Études Cognitives, École Normale Supérieure, EHESS, CNRS, PSL University, Paris, France; 2 Laboratoire de Neurosciences Cognitives Computationnelles, Département d’Études Cognitives, École Normale Supérieure, INSERM, PSL University, Paris, France; 3 Department of Psychology, Royal Holloway University of London, Surrey, United Kingdom; Hebrew University, ISRAEL

## Abstract

Value-based decision-making involves trading off the cost associated with an action against its expected reward. Research has shown that both physical and mental effort constitute such subjective costs, biasing choices away from effortful actions, and discounting the value of obtained rewards. Facing conflicts between competing action alternatives is considered aversive, as recruiting cognitive control to overcome conflict is effortful. Moreover, engaging control to proactively suppress irrelevant information that could conflict with task-relevant information would presumably also be cognitively costly. Yet, it remains unclear whether the cognitive control demands involved in preventing and resolving conflict also constitute costs in value-based decisions. The present study investigated this question by embedding irrelevant distractors (flanker arrows) within a reversal-learning task, with intermixed free and instructed trials. Results showed that participants learned to adapt their free choices to maximize rewards, but were nevertheless biased to follow the suggestions of irrelevant distractors. Thus, the perceived cost of investing cognitive control to suppress an external suggestion could sometimes trump internal value representations. By adapting computational models of reinforcement learning, we assessed the influence of conflict at both the decision and learning stages. Modelling the decision showed that free choices were more biased when participants were less sure about which action was more rewarding. This supports the hypothesis that the costs linked to conflict management were traded off against expected rewards. During the learning phase, we found that learning rates were reduced in instructed, relative to free, choices. Learning rates were further reduced by conflict between an instruction and subjective action values, whereas learning was not robustly influenced by conflict between one’s actions and external distractors. Our results show that the subjective cognitive control costs linked to conflict factor into value-based decision-making, and highlight that different types of conflict may have different effects on learning about action outcomes.

## Author summary

Value-based decision-making involves trading off the cost associated with an action–such as physical or mental effort–against its expected reward. Although facing conflicts between competing action alternatives is considered aversive and effortful, it remains unclear whether conflict also constitutes a cost in value-based decisions. We tested this hypothesis by combining a classic conflict (flanker) task with a reinforcement-learning task. Results showed that participants learned to maximise their earnings, but were nevertheless biased to follow irrelevant suggestions. Computational model-based analyses showed a greater choice bias with more uncertainty about the best action to make, supporting the hypothesis that the costs linked to conflict management were traded off against expected rewards. We additionally found that learning rates were reduced when following instructions, relative to when choosing freely what to do. Learning was further reduced by conflict between instructions and subjective action values. In short, we found that the subjective cognitive control costs linked to conflict factor into value-based decision-making, and that different types of conflict may have different effects on learning about action outcomes.

## Introduction

Voluntary action depends on our capacity to learn how our actions relate to specific events in the external world, and use this knowledge to guide our decisions. Research on value-based decision-making has additionally revealed that the costs associated with specific actions, such as physical [1,2] or mental [3,4] effort, are weighed against their expected rewards [5,6]. In other words, when deciding whether to go out for dinner at a sushi or pizza restaurant, we consider not only how much we like either restaurant, but also how far we need to travel to reach them. Importantly, navigating the external world requires continuously monitoring our decisions, actions, and their consequences, to detect potential difficulties, or unexpected events, that may arise, in order to adapt our behaviour accordingly. Returning to the dinner example, imagine you decide to go to the sushi restaurant but, as you step out of the house, you are faced with the smell of pizza from a new nearby restaurant. This will trigger a conflict between your previous plan to have sushi and the tempting smell of pizza, and may lead you to re-evaluate your decision.

Research on conflict monitoring has shown that detecting conflicts between competing response options leads to the recruitment of cognitive control resources [7,8]. Cognitive control serves to resolve conflict online, for example, by suppressing inappropriate motor activations [9,10], or enhancing attention to task-relevant information [11,12], while sustained adjustments following conflict can help reduce subsequent conflict effects [13–15]. Therefore, cognitive control can be deployed proactively, to prevent or minimise conflict effects, as well as reactively, to resolve conflict once it is detected [16]. However, as engaging cognitive control is effortful, conflict is typically considered aversive [17–19]. When given a choice, people tend to avoid cognitively demanding tasks [20], such as high conflict tasks [21] and contexts [22–24].

Relatedly, many studies have shown that free choices can be biased by external stimuli, whether consciously [25,26] or unconsciously [27–33] perceived. Note that by “free choice” we refer to situations in which the context allows choosing between alternative response options, typically based on internally generated information (e.g. without a reason, based on learned values…). That is contrasted with “instructed” or “forced” choice trials, which are used to refer to situations in which there is only one response option available, i.e. stimulus-driven responses, wherein external information determines the required response given the known rules of the task (e.g. a rightward target arrow requires a right key press). In the aforementioned studies, participants were asked to choose between response alternatives, e.g. pressing a left vs. right button, yet, participants had no particular motivation to pick one action over the other, as they had similar, or no, consequences. Such free choice scenarios have been associated with higher activity in the dorsal anterior cingulate cortex (dACC) than when following instructions [34], with dACC activity also increasing when facing conflict with external stimuli [35,36], in both free and instructed trials [32]. In fact, choosing between indifferent options, or "underdetermined responding" [35], can itself be seen to constitute a type of conflict. When there are no outcomes to motivate the choice (e.g. [32]), or when choice alternatives have similar expected values [37–42], competing responses will be similarly activated, i.e. there will be response conflict. This will require the recruitment of further cognitive resources to break the tie. Importantly, although free choices offer an opportunity to use only *internal* information to guide choice, the presence of additional *external* inputs can trigger activation of one response, e.g. by a left vs. right pointing arrow. Therefore, this context would require the proactive recruitment of cognitive control to prioritise the use of internal and relevant information, by suppressing external distraction, resolving conflict between internal and external information, or delaying the decision. Given the typical tendency to minimise effort and cognitive control engagement, following an external suggestion might then serve to facilitate decision-making. In other words, making use of *any* available information to guide such *inconsequential* decisions would serve to avoid *unjustified* cognitive demands. The choice bias effect could thus be understood as reflecting a similar drive as conflict avoidance, i.e. avoiding cognitive control engagement. Yet, it might seem less clear whether *motivated*, value-based, decision-making would be similarly influenced by *irrelevant*, conscious, external stimuli.

Although the fields of value-based decision-making and conflict monitoring have historically remained largely separate, recent work has started to bridge this gap [7,8,20,36]. For example, Shenhav and colleagues’ [43] Expected Value of Control (EVC) model proposes that the allocation of cognitive control depends on trading off the expected value (i.e. rewards) of control engagement against the amount of control required and its associated cognitive effort costs. In line with this account, cognitive demand avoidance can be modulated by task incentives, and interindividual variability in cognitive control efficiency [20]. Therefore, similarly to how value-based choices are used to infer the subjective (i.e. idiosyncratic) value associated with the choice alternatives, observing the degree to which one’s choices avoid cognitive control demand can be used to infer the subjective costs associated with exerting cognitive control. Shenhav and colleagues’ further proposed that the dACC is a key brain region involved in this cost-benefit analysis [43]. Other neuro-computational models [44–46] have also implicated the dACC in the recruitment of cognitive control resources, monitoring conflict, cognitive and physical effort, difficulty, surprise, or errors, as well as in computing cost-benefit trade-offs that guide the allocation of control. Neuroimaging studies have shown that dACC encodes both mental [3,5] and physical [1,6,47] effort costs during value-based decision-making. Conflict monitoring has also long been associated with dACC activity [8,32,48], further supporting parallels between effort and conflict costs in decision-making [43]. In fact, a recent study showed that interindividual variability in conflict cost was related to its impact on risky decision-making [49]. Following other authors [8,49,50], we will hereafter refer to conflict costs as a shorthand for the aversiveness of the cognitive control demands entailed by conflict situations, including the suppression of irrelevant information and conflict resolution.

Rational, normative accounts of decision-making [51] would predict that decisions with important consequences (e.g. rewards) should motivate us to rely only on relevant, internal information (learned reward expectations), and successfully ignore irrelevant information. Yet, the aforementioned perspective that value-based decisions involve cost-benefit trade-offs would predict that irrelevant information can bias decisions whenever the expected rewards do not outweigh the expected cognitive control costs involved in supressing the irrelevant information. Furthermore, recent work has shown that the competition between top-down vs. bottom-up signals, such as motivation vs. salience, can influence rapid attentional allocation, thus resulting in biases in value-based decisions induced by irrelevant, bottom-up (salience), information [52–55]. That work shows that choice biases can arise from the integration of information from different sources, given the choice context and input, leading to facilitation of a given response by irrelevant information, rather than invoking a role for conflict management (cf. [54]). While not necessarily being inconsistent with such facilitation mechanisms, the perspective that people are motivated to avoid cognitive demands can offer an explanation as to why the recruitment of cognitive control resources is not enhanced to prevent such biases in the first place, e.g. proactively, whenever the expected control costs seem unjustified by the expected benefits. Notably, this perspective also sheds light on the observation that, although free choices are typically preferred over no choice, the difficulty of the choice context, such as when making choices under uncertainty, or when having many options to consider (aka. choice overload [56]), can render free choice undesirable [57], and reduce the subjective freedom experienced [58]. Therefore, our study aimed to further investigate this cost-benefit trade-off by investigating whether biases in free choices induced by conscious distractors (flankers) would be evident in a value-based context, similarly to had been previously observed for “indifferent” choices [26].

Independently of how conflict costs factor into our *decision-making*, experiencing conflict during a decision could also alter how we *learn* about action-outcomes associations, i.e. instrumental learning. The aversive nature of conflict has been shown to influence the processing of action outcomes. Conflicts can lead to a more negative evaluation of neutral stimuli [59,60], and a reduction in perceived control over action outcomes [61,62]. In line with findings on effort discounting [4,6], a recent study showed that response conflict may carry an implicit cost to obtained rewards [50]. Using the Simon task [63], Cavanagh and colleagues showed that participants preferred cue stimuli associated with rewards that followed non-conflicted trials, over stimuli associated with rewards that followed conflicted trials. Importantly, during the learning phase of that study, participants could not choose what to do (i.e. they had to follow an instruction in the stimulus), hence could not make an action that did not trigger conflict. Yet, from the perspective that the allocation of cognitive control depends on cost-benefit analyses, in a free choice scenario in which an available response option could serve to avoid or minimise conflict, e.g. by choosing an easier task or response, choosing the option that does entail conflict would likely be motivated by lower conflict costs or higher reward expectations. In line with a moderating role for freedom of choice for conflict costs, choosing freely to do a cognitively demanding task (high conflict probability) was linked to greater striatum activity than when choosing the easy task, implying an intrinsic motivation that offset the cognitive control costs, whereas striatum activity patterns were reversed when having to follow instructions [22]. Therefore, it remains unclear whether conflict costs would still influence learning when participants *could* have made a choice that would not entail conflict.

Finally, in addition to experiencing conflicts between internal and external information–akin to "pizza smell" example above, we can also experience conflicts between competing internal motivations–e.g. preferring sushi, but also wanting to please a friend who asks to have pizza. Interestingly, it has been shown that motivational conflicts, such as between Pavlovian biases and instrumental task requirements, can impair instrumental learning [64,65]. This work shows that it is difficult to learn to act to avoid punishments, as it goes against the Pavlovian tendency of withholding action to avoid punishments. The competing motivations will thus activate competing response options, requiring cognitive control to suppress the inappropriate Pavlovian bias [66]. Despite the differences in the underlying sources of conflict, common neural signals have been implicated in monitoring externally-triggered and motivational conflicts [66,67] (i.e. mid-frontal theta band oscillations, in turn thought to be linked to ACC [68]). These findings further support the hypothesis that conflict costs could alter learning. Nonetheless, it remains possible that the precise nature of the conflict experienced–between internal vs. external information, or between competing internal motivations–could be a relevant moderator of its effects on learning.

**Fig 1:**
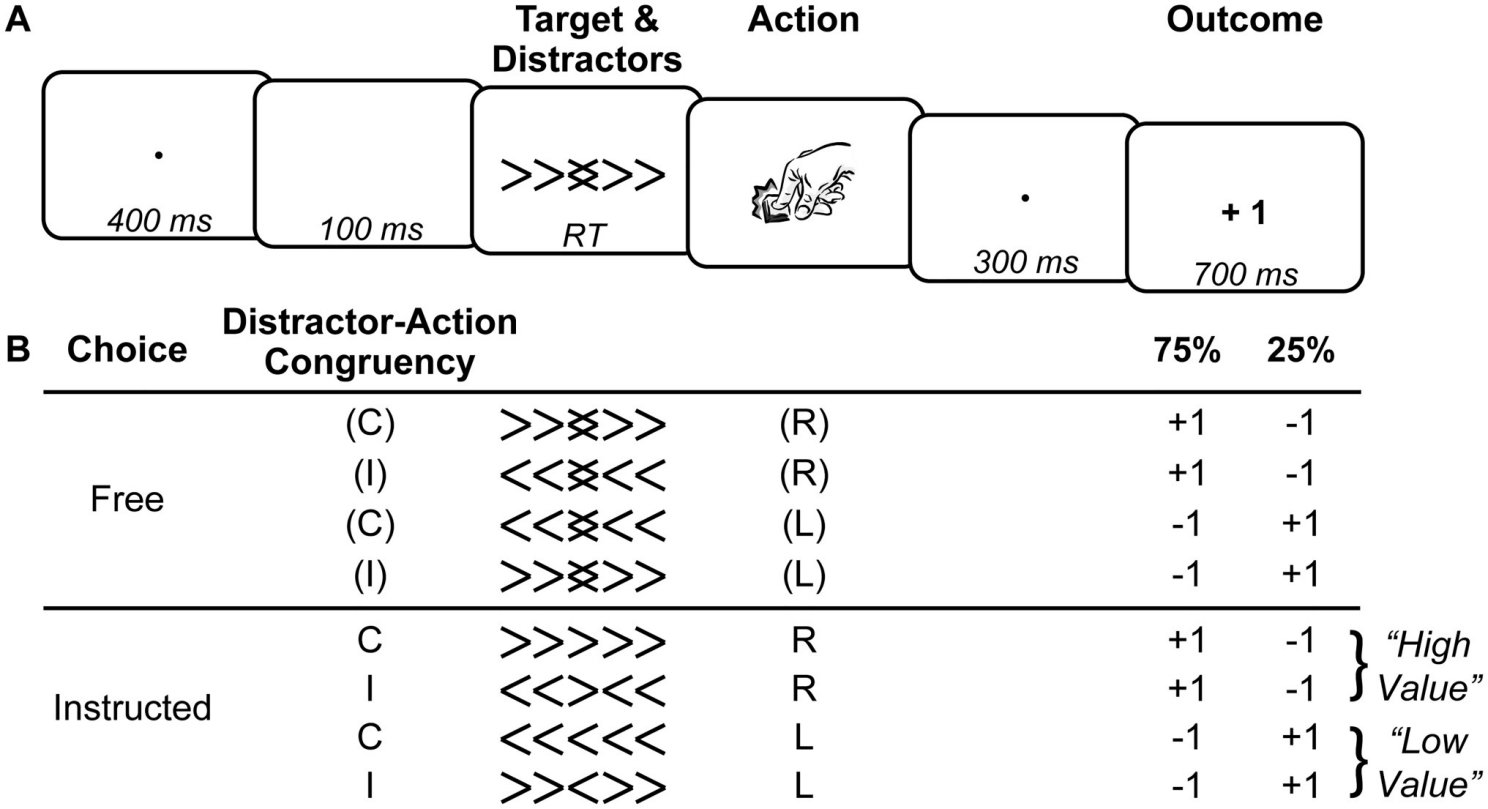
Task outline. **A.** Timeline of a trial. **B.** Task design and example mapping of actions to reward probabilities. Conflict between actions and external distractors is captured by the "distractor-action congruency" factor, where C = Congruent, and I = Incongruent. Conflict between instructions and subjective action values (model-based) is exemplified here. Assuming participants correctly learned the current contingency, right (R) would be the subjectively "high value" action (i.e. no conflict if instructed right), and left (L) would be the "low value" action (i.e. conflict if instructed left).

The present study aimed to investigate the following two key questions: a) whether value-based decisions could be influenced by irrelevant distractors; b) whether experiencing conflict might influence instrumental learning. Additionally, we assessed the role of two potential moderators of how learning might be influenced by conflict: i) the type of conflict experienced–with external information, or between internal motivations; ii) choice freedom, since having the possibility to make choices that could reduce conflict might alter the experience of conflict when the difficult option is chosen (in a free choice scenario), relative to when conflict is unavoidable (when following instructions). To test these questions, we embedded irrelevant distractors (flankers) within a reversal-learning task (Fig 1), with intermixed free and instructed trials. Participants had to continuously track whether left or right hand actions had a high or low reward probability (75/25%), and contingencies reversed unpredictably. As the same contingencies applied in free and instructed trials, participants were told to learn equally from the outcomes of both trial types, and that not complying with instructions would reduce their final earnings. Distractors could trigger conflict with an instructed action (e.g. >><>>) or with a freely chosen action (indicated by a bidirectional target), and might bias free choices. In this context, participants could adapt to conflict by focusing on the target and ignoring the distractors, while free choices additionally offered an opportunity for conflict avoidance. Comparing the influence of conflict on learning in free and instructed trials allowed us to assess the role of having choice in whether to act in conflict with an external suggestion. Furthermore, as instructions were equally likely to require making the high or low reward action, participants sometimes experienced conflict between two internal motivations: correctly following an instruction (e.g. left), and following their subjective value expectations about the best action (e.g. right).

As brief glimpse of our findings, computational models of reinforcement learning [69,70] were adapted to test our hypotheses, and fitted to trial-by-trial choice behaviour. Model comparison showed that a model implementing a distractor bias in the decision rule outperformed a simple RL model in describing the data. The data and the model supported our hypothesis that value-based choices could be biased by irrelevant information, as conflict costs were traded off against expected rewards. Comparing models with different learning rates showed that learning was influenced by freedom of choice, and by conflict in instructed trials, when facing a motivational conflict between the instruction and subjective action values.

## Results

### Distractor effects on action

To verify that the distractors (i.e. flankers) elicited response conflict we analysed the effect of distractor congruency on different behavioural variables: reaction times, free choices, and error rates.

#### Reaction times

**Fig 2:**
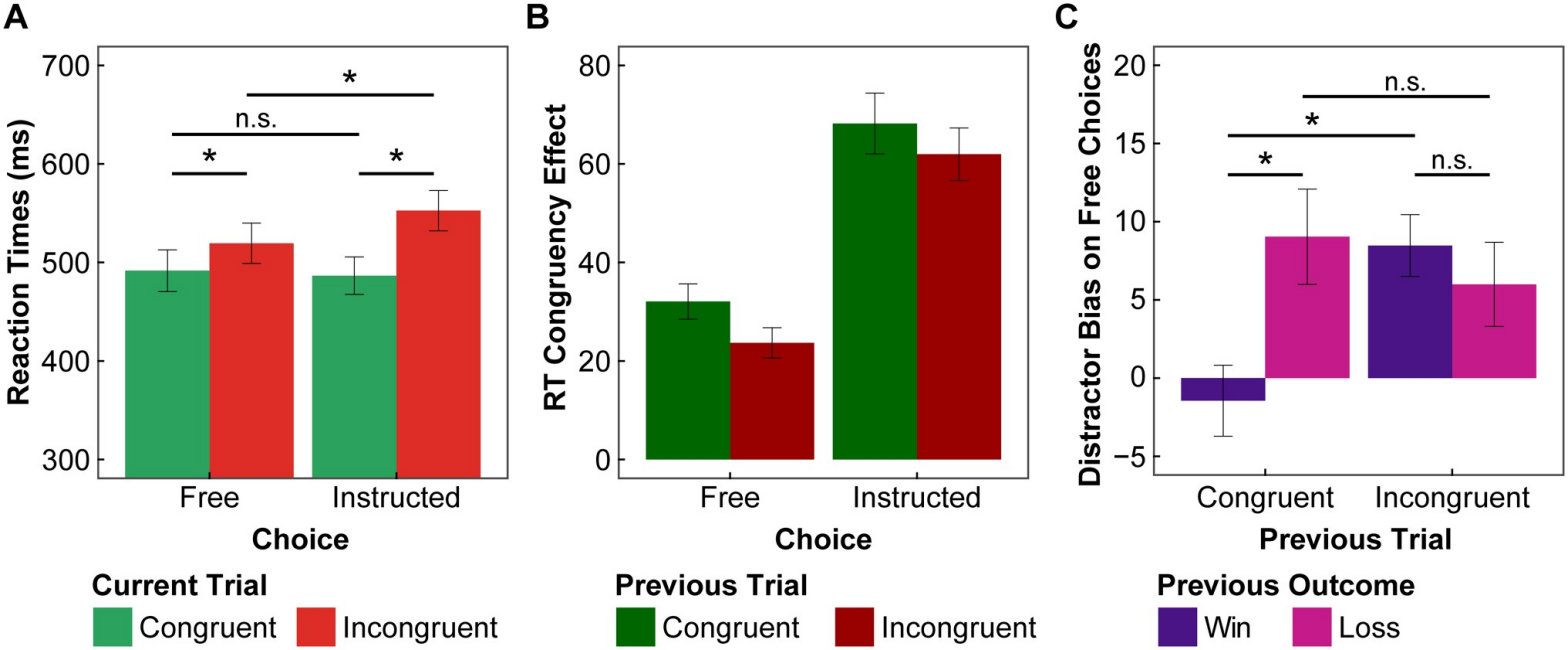
Influence of conflict at the decision stage. **A.** Average RTs as a function of choice and current trial distractor-action congruency. **B.** Average conflict effects on RTs (incongruent *minus* congruent) as a function of choice and previous trial congruency. **C.** Average distractor bias effect on free choices (percentage of congruent *minus* incongruent choices) as a function of previous trial congruency and outcome (win/loss). Error bars represent the standard error of the mean. * p < .05, n.s. = non significant.

Mean reaction times (Fig 2A) were submitted to a repeated-measures ANOVA, as function of choice (free vs. instructed), and current distractor-action congruency (congruent vs. incongruent). This revealed a significant main effect of distractor-action congruency (*F*_1,19_ = 182.29, *p* < .001, ${ƞ}_{p}^{2}=0.91$), as well as a significant main effect of choice (*F*_1,19_ = 7.52, *p* = 0.01, ${ƞ}_{p}^{2}$ = 0.28), and a significant choice-by-congruency interaction (*F*_1,19_ = 59.78, *p* < .001, ${ƞ}_{p}^{2}=0.76$). Post-hoc tests revealed that there was a significant congruency effect in both free and instructed trials (free: *t*_19_ = -10.60, *p* < .001, *d* = -2.38; instructed: *t*_19_ = -12.10, *p* < .001, *d* = -2.72), with slower RTs in incongruent than congruent trials. Additionally, for incongruent trials, RTs in instructed trials were significant slower than in free trials (*t*_19_ = -7.10, *p* < .001, *d* = -1.57), but there was no significant effect of choice in congruent trials (*t*_19_ = 0.78, *p* = .45, *d* = 0.17). The same findings were observed with log-transformed RTs. These findings show that choices that went against the distractors’ suggestion carried a cost to performance (i.e. slower RTs in free-incongruent than in free-congruent trials). Moreover, the cost of conflict to action selection was even greater in instructed trials (i.e. slowest RTs in instructed-incongruent trials). To correctly follow the instruction in incongruent trials, participants had to overcome conflict both at the level of the visual stimuli (to detect the target direction among opposing distractors), and of the response (as distractors and target triggered two competing responses).

#### Free choices

In free trials, participants were significantly biased towards choosing actions that were congruent with the direction of the distractors, rather than making distractor-incongruent actions (proportion congruent: 52.70% ±4.57; one sample t-test against 50% chance level: *t*_19_ = 2.65, *p* = .016, *d* = 0.84).

#### Errors

In instructed trials, participants made significantly more errors, i.e. not responding according to the target direction, when the distractors were incongruent with the target direction, than in congruent trials (congruent: 1.30% ±1.59; incongruent: 4.58% ±2.58; paired t-test: *t*_19_ = -5.78, *p* < .001, *d* = -1.29). This shows the strength of the disruption of the distractors to action selection, occasionally even leading participants to make the wrong action.

### Sequential conflict adjustments

Previous work with classic conflict tasks has revealed different types of behavioural adjustments following conflict, whether through conflict adaptation–reflected in reduced conflict effects on RTs (the “Gratton effect”, [15]), or through conflict avoidance–reflected in higher proportion of choices for easy (low-conflict) options, and both strategies can be used simultaneously (e.g. [21]). Therefore, we sought to explore whether similar sequential adjustments to conflict might also be observed within the context of our reinforcement learning task.

Regarding conflict adaptation on RTs, in addition to testing whether it could be observed in our study, we also explored whether it depended on whether the current trial was free or instructed. Current trial congruency effects on RTs (incongruent *minus* congruent) were assessed as a function of choice (free vs. instructed) and previous trial congruency (congruent vs. incongruent; see Fig 2B). Repeated-measures ANOVA revealed a significant main effect of choice (*F*_1,19_ = 58.82, *p* < .001, ${ƞ}_{p}^{2}=0.76$), indicating that congruency effects were overall larger in instructed than in free trials, in line with the previous findings on RTs. Additionally, there was a significant main effect of previous trial congruency (*F*_1,19_ = 5.30, *p* = .03, ${ƞ}_{p}^{2}=0.22$), indicating conflict adaptation, as congruency effects on the current trial were reduced following incongruent trials, relative to following congruent trials. There was no significant interaction between the factors (*F*_1,19_ = 0.19, *p* = .67, ${ƞ}_{p}^{2}=0.01$), suggesting a similar conflict adaptation regardless of whether the current trial was free or instructed. As before, the same findings were observed with log-transformed RTs.

Next, we assessed whether the biasing effect of the distractors on free choices was affected by both conflict and reward history (Fig 2C). The previously observed conflict avoidance effects would here be reflected in a greater choice bias, which might be increased following conflict. Moreover, we reasoned that the outcome in the previous trial could also modulate the cost-benefit trade-offs in the decision, as it could change the expected action values. We computed a "distractor bias" measure (percentage congruent *minus* incongruent free choices), which denoted the degree to which participants followed the distractors’ suggestion. This "distractor bias" variable was submitted to a repeated-measures ANOVA with previous trial congruency and previous outcome (win vs. loss) as within-subject factors. Results showed a significant main effect of previous trial congruency (*F*_1,19_ = 5.49, *p* = .03, ${ƞ}_{p}^{2}=0.22$), a significant main effect of previous outcome (*F*_1,19_ = 4.79, *p* = .04, ${ƞ}_{p}^{2}=0.20$), and a significant interaction between the two factors (*F*_1,19_ = 10.31, *p* = .005, ${ƞ}_{p}^{2}=0.35$). Post-hoc tests revealed that the distractor bias was significantly larger after loss than win outcomes following congruent trials (*t*_19_ = -3.84, *p* = .001, *d* = -0.86), however there was no effect of previous outcome following incongruent trials (*t*_19_ = 0.91, *p* = .37, *d* = 0.20). At the same time, the distractor bias was larger after incongruent, than congruent, trials following win outcomes (*t*_19_ = -5.01, *p* < .001, *d* = -1.12), whereas there was no effect of previous trial congruency following loss outcomes (*t*_19_ = 1.04, *p* = .31, *d* = 0.23). That is, if the previous trial was incongruent, or resulted in a loss, participants were more likely to be biased by the distractors’ suggestion.

In line with previous accounts (e.g. [8,21]), we speculate that recent conflict experience might increase the saliency of conflict costs, while losses might reduce the participant’s confidence in their knowledge of the best response, i.e. reducing expected rewards. These aversive experiences would shift the cost-benefit trade-off in the following trial towards a greater choice bias, presumably due to increased avoidance of cognitive control. Only when the last trial involved easy action selection and was rewarded (i.e. all "went well") were participants *not* biased by the distractors (Fig 2C, one sample t-test of distractor bias in “previously congruent and win outcome” trials against 0 confirms there was no significant difference, *t*_19_ = -0.64, *p* = .53, *d* = -0.20; whereas distractor bias was larger than 0 in the remaining trial types, all *t*s > 2.22, *p*s < .039, *d* > 0.70). That is, the absence of any challenge in the preceding trial might have increased participants’ willingness to invest cognitive control to suppress the distractor influence and rely more on their internal action values.

It is worth bearing in mind that these analyses remain exploratory. Since sequential conflict effects were not our primary interest, our task was not designed to optimise those analyses. Notably, we recognise that other factors that varied across trials might also dynamically influence conflict adjustments, such as whether there were repetitions in the executed action, flanker direction, or choice condition. However, due to considerable number of factors in our task, considering too many factors in the previous vs. current trial could easily lead to high-order interactions that would not be interpretable. Hence, our analyses focused on testing specific hypothesis derived from previous work and experience with task.

### Learning performance

**Fig 3:**
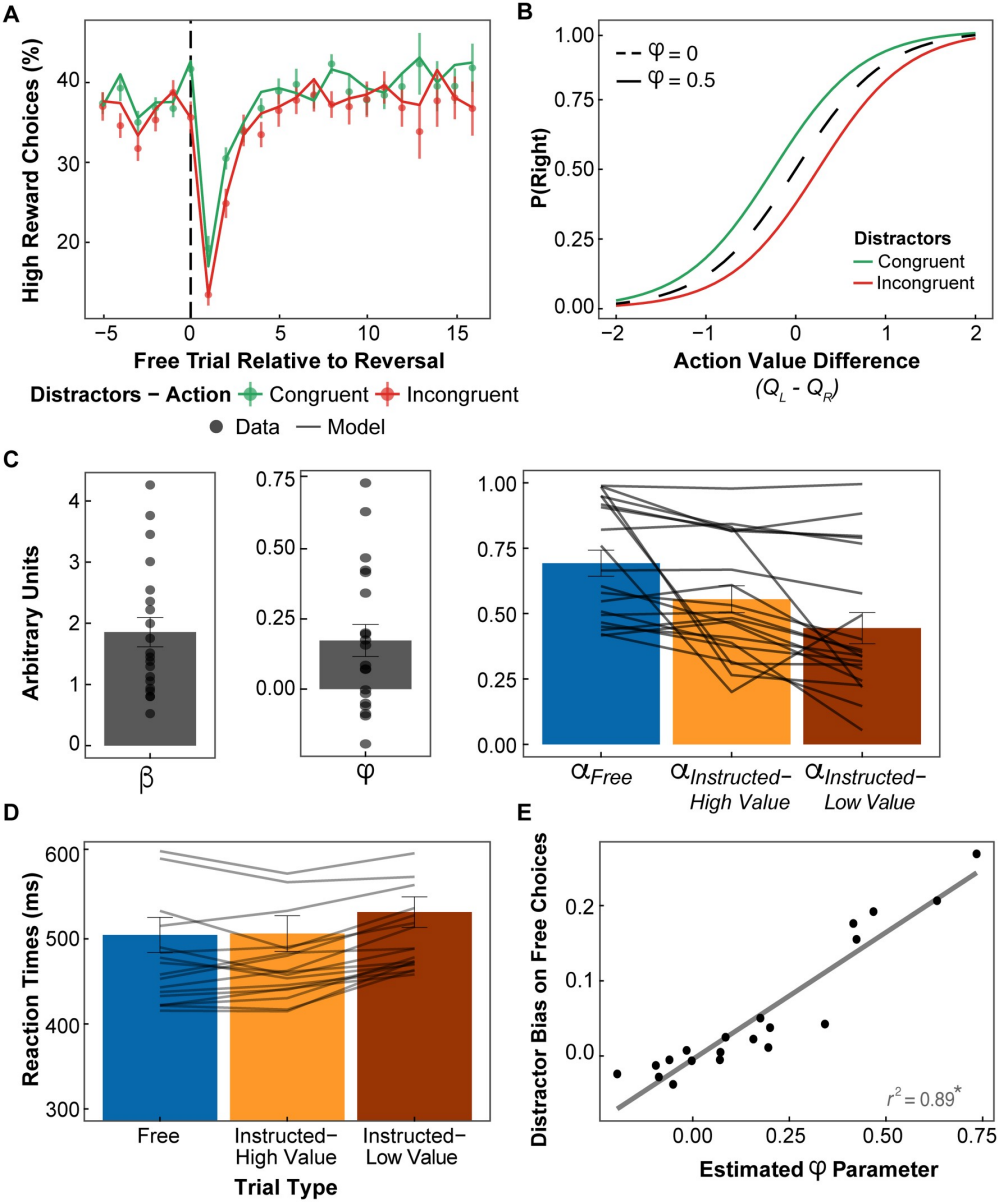
Model-based analyses of reinforcement learning (for wining model: M8). **A.** Learning curve (real data as points with standard errors, and model simulations as lines) displaying the percentage of high reward choices, and whether they were congruent or incongruent with the distractors, as a function of free trial number relative to a reversal point. **B.** Hypothetical softmax decision rule (with the choice temperature parameter *β* = 2) for hypothetical distractor bias parameters (*φ* = 0 vs. *φ* = 0.5), and as a function of distractor-action congruency (for *φ* = 0.5). **C.** Average estimated parameters, where *β* = choice temperature, *φ* = distractor bias, and *α* = learning rate (as bars; with dots/lines representing each participant). **D.** Average RTs a function of choice, and whether instructions required making the subjectively high or low value action (based on simulated action (*Q*) values; lines represent each participant). **E.** Relation between the estimated distractor bias parameter (*φ*) and the observed distractor bias on free choices.

To assess whether participants were able to accurately learn to make the best response, we calculated the percentage of high reward choices as a function of free trial number (i.e. ignoring instructed trials) relative to a reversal point. Given the choice bias identified above, we additionally considered how choices were affected by distractor-action congruency, and thus calculated the percentage of congruent or incongruent high reward choices, relative to the total number of free trials. The resulting learning curves (Fig 3A) show that participants successfully learned to maximise their rewards over time, matching objective reward probability level (75%, shown in Fig 3A divided by the 2 congruency conditions, i.e. 37.5%). In the free choice before a reversal, participants chose the high reward option on 76.13% ±0.09 of trials (one sample t-test against 50% chance level: *t*_19_ = 13.63, *p* < .001, *d* = 4.31). The learning curves additionally show that free choices were biased by the distractors, as participants were more likely to make congruent than incongruent choices, particularly at the start of learning episodes.

### Modelling the effects of conflict between action and distractors

Computational models of reinforcement learning [69,70] were adapted to better investigate our two research questions, i.e. the effects of conflict with external distractors on a) decisions, and b) learning (see Materials and methods below for further details). Models were fitted to participants’ choices. At the decision stage, to capture the distractor bias on free choices described above, we adapted a standard softmax decision rule to include a distractor bias parameter, *φ*. The softmax rule serves to estimates the probability of selecting a particular option (e.g. right) as a sigmoid function of the difference in action values (left vs. right), defined by a temperature parameter (*β*), which captures choice stochasticity. The added distractor bias parameter can shift the softmax curve to increase the probability of making distractor-congruent choices (if *φ* > 0, see Fig 3B). This distractor bias parameter (*φ*) captures the degree to which participants’ free choices are biased by the distractors, such that a greater difference in proportion of congruent vs. incongruent choices would be related to a larger distractor bias parameter (as was indeed observed, see Fig 3E). From the perspective that the cognitive control demands involved in preventing and resolving conflict carry a subjective cost, a larger choice bias would reflect a greater motivation to avoid conflict. Consequently, the distractor bias term could be understood as an implicit measure of the subjective cost of conflict, i.e. how much of an increase in expected rewards is required to offset the costs of cognitive control. At the learning stage, we used a standard *Q*-learning model to track how the expected action values (*Q*-values) are updated across trials. To test the effect of action-distractor conflict on learning, we compared models with separate learning rates (*α*) for congruent vs. incongruent trials, as well as a function of choice and choice-by-congruency interactions.

**Fig 4:**
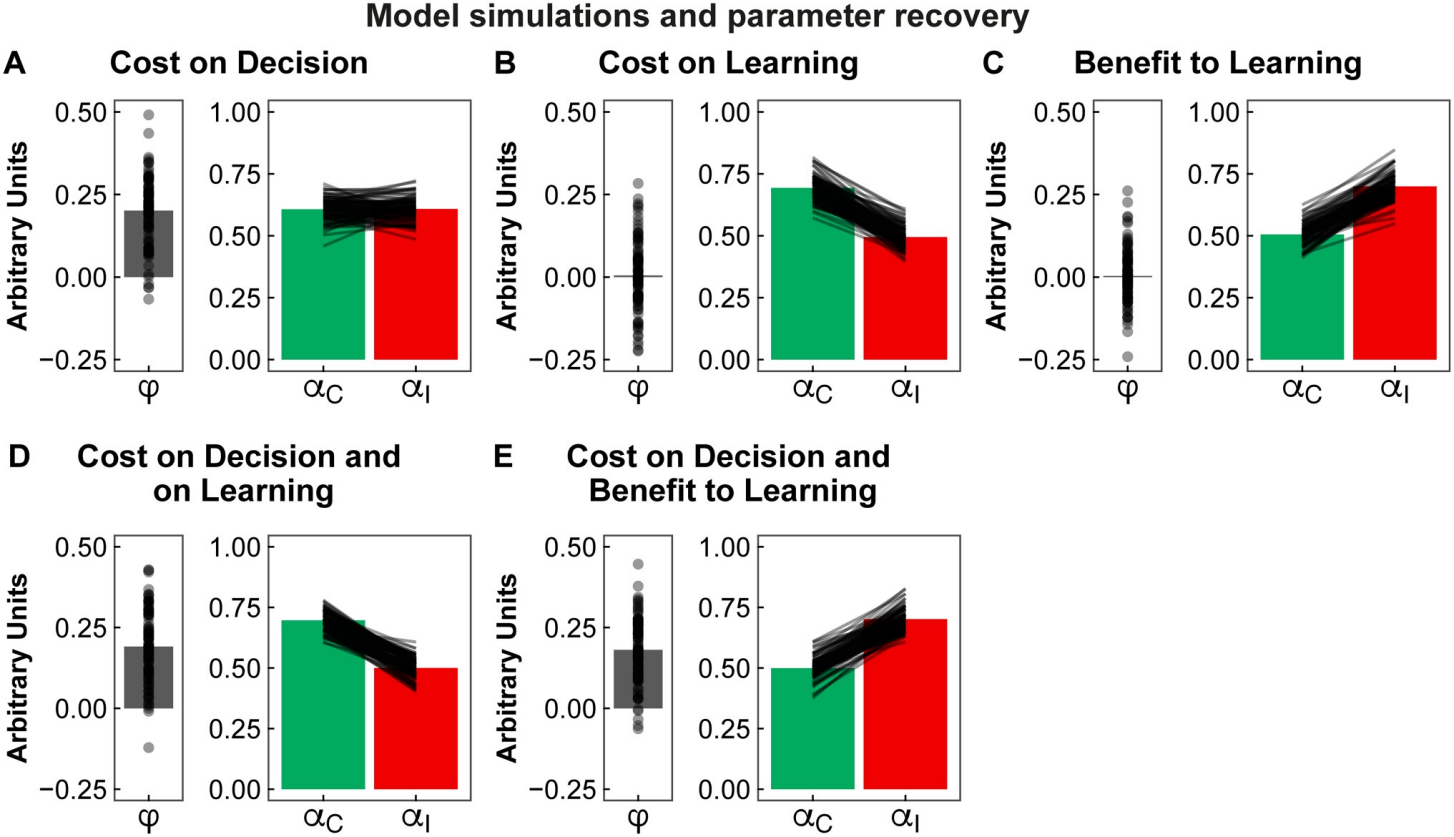
Parameter recovery validation. Validation of the parameter optimisation procedure, and of the ability of our model to capture dissociable effects of action-distractor conflict at the decision and learning stages. Virtual data (*N* = 100, displayed as dots and lines) was simulated based on five different parameter sets (while holding the choice temperature parameter, *β* = 2). The bars display the average parameters obtained from applying the parameter optimisation procedure. A. Results from data simulated with a cost of conflict on the decision [*φ* = 0.2, *α*_*C*_ = *α*_*I*_ = 0.6]. B. Results for data simulated with a cost of conflict on learning [*φ* = 0, *α*_*C*_ = 0.7, *α*_*I*_ = 0.5]. C. Results for data simulated with a benefit of conflict to learning [*φ* = 0, *α*_*C*_ = 0.5, *α*_*I*_ = 0.7]. Panels D. and E. combine the effect on the decision show in A, with the effects on learning in B and C, respectively. The results confirm that the simulated model parameters are adequately recovered by the optimisation procedure, as well as the dissociability of effects on the decision (*φ*) from effects on learning (*α*). *φ* = distractor bias, *α* = learning rate, C = Congruent, I = Incongruent.

We tested the ability of our computational model and task to capture dissociable effects of action-distractor conflict on the decision (*φ*) and on learning (*α*) by simulating virtual data with different hypothetical effects (Fig 4, and see “Validation of the model and parameter optimisation” section, in Methods, for details). These simulated independent possible costs of conflict on the decision (Fig 4A) or on learning (Fig 4B), as well as combined (Fig 4C and 4D) or even opposing effects (i.e. cost to decision, but benefit to learning, Fig 4E). This confirmed that any observations about the location of distractor conflict costs at the decision vs. learning stages were not confounded, or biased, by an inherent feature of our modelling procedure or of our task design.

Turning to the modelling of the behavioural data, we initially compared a classic reinforcement learning model with one choice temperature (*β*) and only one learning rate (*α*), with models that additionally included our distractor bias (*φ*) parameter, and potentially separate learning rates as a function of choice and congruency. Not including the model that considered conflict between instructions and action values (described in the next section) in this initial model space allowed us to test whether there was *any* effect of action-distractor conflict on learning, rather than compare which type of conflict effects on learning might better fit the data. Model comparison (S1 Table) revealed that the winning model (m4) included the distractor bias (*φ*) parameter in the decision rule, and different learning rates as function of choice only (*xp = 0*.*97*). For this model, and in line with the observed choice bias towards distractor-congruent choices, we found that the estimated distractor bias parameter was significantly larger than 0 (average *φ* = 0.17 ± 0.25; one-sample t-test against 0: *t*_19_ = 3.05, *p* = .007, *d* = 0.97). Turning to the learning rates, a paired sample t-test revealed that learning rates were significantly higher in free than in instructed trials (*t*_19_ = 3.93, *p* < .001, *d* = 0.88).

Since the models with different learning rates as a function of congruency did not provide a significantly better fit to the data (considering the number of free parameters), we conclude that the present data did not reveal robust effects on learning of action-distractor conflict (see S1 Text for further analyses).

### Modelling the effects of conflict between instructions and subjective action values

To assess whether learning was influenced by motivational conflict between instructions and subjective action values, we considered an additional model (m8). Instruction-value conflict trials require resolving a conflict between two internal drives: correctly following the instruction vs. choosing the most rewarding option. Since errors in instructed trials reduced participants’ final earnings, they were still motivated to correctly respond according to the instruction. The extra model (m8) parses learning rates in instructed trials as a function of estimated action values (*Q*-values): if the instruction required the action favoured by the difference in action values, the trial was classed as “Instructed-High Value”, otherwise it was classed as “Instructed-Low Value”. As the previous model comparison showed an influence of choice on learning rates, this new model also allowed us to test whether the reduction in learning rates in instructed trials was specifically driven by trials in which participants had to follow an instruction that went against their subjective action values (i.e. instructed-low value).

Comparing this with all previously considered models allowed us to check the winning model provided the best fit to the data across the model space. Moreover, as the previous model comparison did not reveal robust effects of action-distractor conflict on learning, we did not additionally include models with both types of conflict. Model comparison across this extended model space (Table 1) showed that the new model (m8) provided a better fit to the data than all other models (*xp* = 0.97), including the previously winning model (m4). This confirmed that instruction-value conflict had a robust influence on learning rates.

**Table 1:** Model comparison across the extended model space.

Models	AIC ± SD	Model Frequency	Exceedance Probability (*xp*)
m1: Standard RL [*β*, *α*]	723.3 ± 191.4	0.15	0.02
m2: [*β*, *φ*, *α*]	714.3 ± 183.5	0.08	0.00
m3: [*β*, *φ*, *α*_*C*_ ≠ *α*_*I*_]	715.2 ± 184.0	0.04	0.00
m4: [*β*, *φ*, *α*_*Free*_ ≠ *α*_*Instructed*_]	704.7 ± 176.8	0.08	0.00
m5: [*β*, *φ*, *α*_*Free_C*_ ≠ *α*_*Free_I*_ ≠ *α*_*Instructed*_]	704.9 ± 178.4	0.06	0.00
m6: [*β*, *φ*, *α*_*Free*_ ≠ *α*_*Instructed_C*_ ≠ *α*_*Instructed_I*_]	705.2 ±177.4	0.05	0.00
m7: [*β*, *φ*, *α*_*Free_C*_ ≠ *α*_*Free_I*_ ≠ *α*_*Instructed_C*_ ≠ *α*_*Instructed_I*_]	705.4 ± 178.8	0.10	0.00
**m8: [*β*, *φ*, *α***_***Free***_ **≠ *α***_***Instructed_High Value***_ **≠ *α***_***Instructed_Low Value***_**]**	**701.8 ± 177.8**	**0.44**	**0.97**

*β* refers to choice temperature parameter, reflecting choice stochasticity. *φ* refers to the distractor bias parameter added to the decision rule. α refers to the learning rates parameters, split by conditions. RL = Reinforcement Learning, C = Congruent, I = Incongruent, AIC = Akaike Information Criteria, SD = Standard Deviation.

While model comparisons are important to determine which model best fits the data, i.e. assessing the model’s *predictive performance*, it is also vital to verify that the winning model can adequately replicate participants’ behaviour [71], i.e. assessing the model’s *generative performance*, through model simulations. Based on the average estimated parameter values for the winning model (m8), we simulated data across the participants’ trial sequences. The simulation results can be observed in Fig 3A, together with the real participants’ behaviour. This demonstrates that our model was indeed able to replicate critical aspects of participants’ behaviour, such as the distractor bias on free choices (see S2 Text for further simulations demonstrating that including the distractor bias (*φ*) parameter in the decision rule is essential to capturing this effect).

### Effects of choice and instruction-value conflict on learning

To better understand how choice and instruction-value conflict influenced learning, we submitted the estimated learning rates (Fig 3C) to a one-way repeated-measures ANOVA based on trial type (free, instructed-high value, instructed-low value). As expected, this showed a significant effect of trial type (*F*_1.84, 34.87_ = 13.72, *p* < .001, ${ƞ}_{p}^{2}=0.42$). Bonferroni-corrected follow-up tests confirmed that instruction-value conflict led to a significant reduction in learning rates (instructed high vs. low value: *t*_19_ = 2.78, *p* = .036, *d* = 0.62). Moreover, the results showed that learning rates were significantly reduced by following instructions, even when the instruction required making what was believed to be the best action (free vs. instructed-high value: *t*_19_ = 2.70, *p* = .043, *d* = 0.60; free vs. instructed-low value: *t*_19_ = 4.89, *p* < .001, *d* = 1.09). This shows that the reduction in learning rates in instructed than free trials seen in the previous simpler model (m4) cannot be fully explained by trials in which participants were instructed to go against their beliefs.

#### Effects of choice and instruction-value conflict on action selection

Next, we assessed whether changes in action selection (RTs), as a proxy for conflict, were associated with the observed differences in learning rates. For this, we used the parameters estimated from the winning model (m8) for each participant to simulate action values (Q values) for each trial. This allows us to categorise instructed trials as high or low value, and thus extract the participant’s real RTs (Fig 3D) associated with each trial type (free, instructed-high value, instructed-low value). One-way repeated-measures ANOVA showed a significant main effect of trial type (*F*_1.82, 34.58_ = 15.59, *p* < .001, ${ƞ}_{p}^{2}=0.45$). Bonferroni-corrected follow-up tests showed that instruction-value conflict led to significantly slower RTs than no conflict (instructed high vs. low value: *t*_19_ = -5.26, *p* < .001, *d* = -1.18), or free choice (free vs. instructed-low value: *t*_19_ = -4.36, *p* = .001, *d* = -0.98). This confirms that the cost of motivational conflict, between instructions and action values, was evident in participants’ behaviour, and might therefore be related to the associated reduction in learning rates.

Turning to the comparison of free choice vs. instructed-high value trials, one could have hypothesised that the observed reduction in learning rates could also be related to cognitive control costs. When participants detected an instruction target, they could have suppressed any advanced action preparation based on expected values, to more accurately process the stimuli. However, there was no significant difference in RTs between free choice and instructed-high value trials (free vs. instructed-high value: *t*_19_ = -0.34, *p* = 1.00, *d* = -0.08). This suggests that cognitive control costs might not explain the reduction in learning rates observed when participants followed an instruction they agreed with.

Taken together, these results further suggest that participants only recruited cognitive control when needed. If the instruction allowed it, participants went ahead with the prepared action (same RTs as free trials), and only had to suppress it if conflicted with the instruction (slower RTs in instructed-low value trials). This view was further supported by supplementary analyses of action selection (RTs and ERs) in instructed trials as a function of both instruction-value and action-distractor conflict (S4 Text).

### Relations between distractor bias parameter and behaviour

The estimated distractor bias in the new model (m8) was virtually identical to that estimated in previously winning model (average *φ* = 0.17 ± 0.26; one-sample t-test against 0: *t*_19_ = 3.03, *p* = .007, *d* = 0.96). To confirm that the estimated parameter was indeed related to participants’ choice bias, as expected, we assessed the correlation between the parameter estimates and the average distractor bias measure on free choices (percentage congruent *minus* incongruent). This confirmed a highly significant correlation (see Fig 3E; Pearson’s correlation: *r* = 0.94, *t*_18_ = 12.18, *p* < .001). [See S3 Text for further correlations between the distractor bias parameter and behaviour, and simulations demonstrating that the distractor bias did not robustly disrupt task performance.]

## Discussion

**Fig 5:**
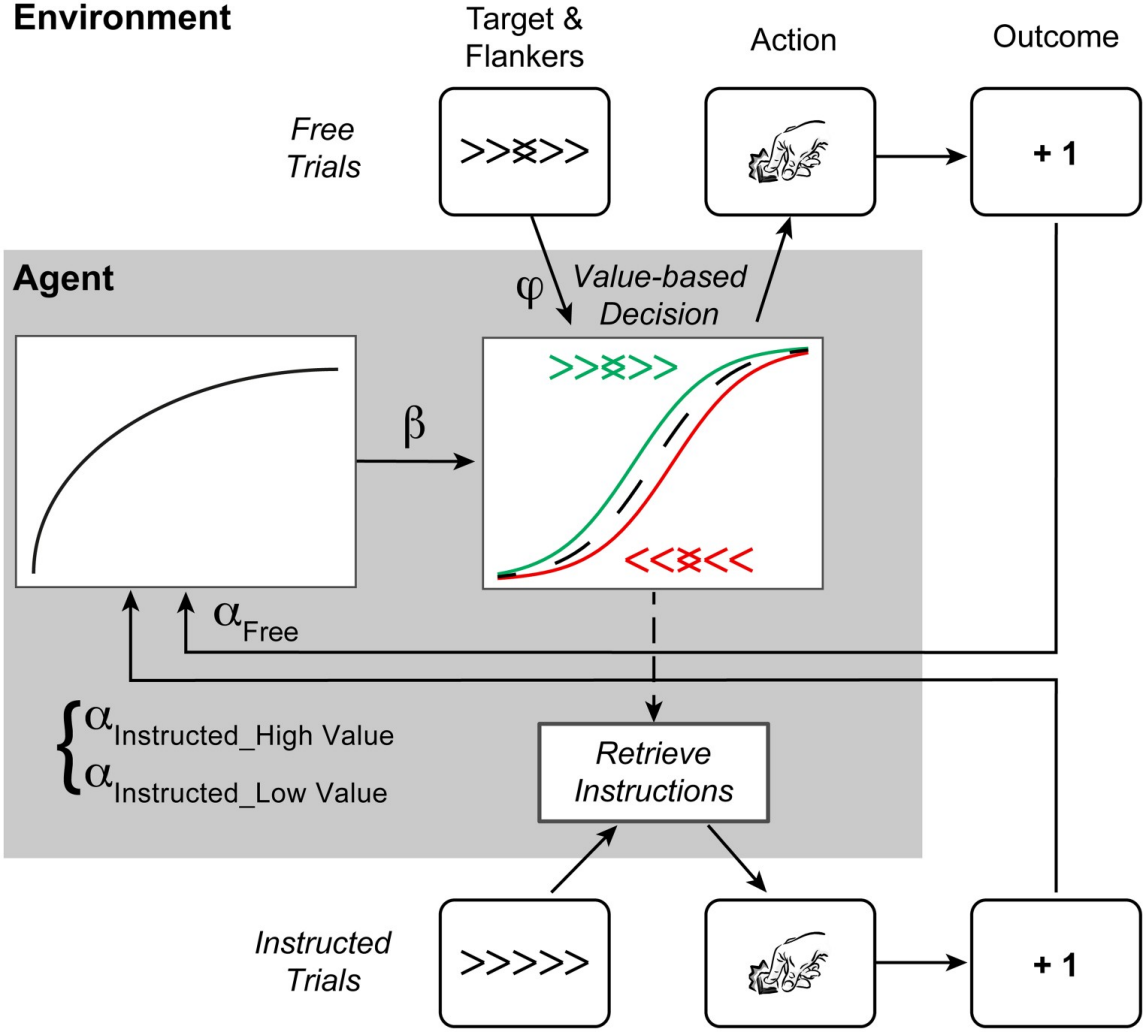
Schematic representation of the winning computational model. In Free trials, participants make value-based decisions by integrating internal information about action values (as a function of the temperature parameter, *β*) with the external suggestion of the distractors (as a function of the distractor bias parameter, *φ*). The distractor bias parameter captures the increased the likelihood that participants will choose actions that are congruent with the distractors, especially when the difference in action values is small (i.e. larger gap between green/red lines in the middle of the graph of the value-based decision, see also Fig 3B). This can be interpreted as reflecting the participants’ tendency to avoid conflict, and its associated cognitive control demands, especially when the expected benefits are low. The outcomes of those freely chosen actions can then be used to update action values (as a function of the learning rate, *α*_*Free*_). In instructed trials, participants must retrieve the instruction of following the target direction, but the accumulated action value information may partially interfere with their responses (influence on RTs seen in Fig 3D). Moreover, action outcomes are used to update actions values differently depending on whether the instruction required making the subjectively high vs. low value action (*α*_*Instructed_High Value*_ vs. *α*_*Instructed_Low Value*_ respectively, based on the previously accumulated values).

The present study investigated the influence of response conflict on value-based decision-making and learning. For this, we combined a flanker task, in which distracting stimuli could trigger response conflict, with a reversal-learning task, requiring the learning of action-outcome associations. Our results show that even *motivated*–value-based–decision-making can be biased by *irrelevant* external information. Our findings, summarised in Fig 5, suggest that this bias results from a trade-off between the expected value of a given action and the cognitive control costs involved in handling potential conflict with an external suggestion. At the learning stage, we found that participants updated their value representations less when they had to follow instructions than when they could freely choose what to do. Learning was further reduced when participants had to follow instructions that went against their subjective beliefs. Thus, we found that learning was influenced by conflict between instructions and subjective action values, but did not find robust evidence that learning was influenced by conflict between one’s actions and external distractors.

### Influences on decision-making

At the decision stage, we found that decisions guided by internal value representations could be biased by external information. When free to choose what to do, participants typically chose the most rewarding action. Nevertheless, participants were still more likely to choose an action that was congruent with the suggestion of distracting stimuli, than to choose the opposite action. Moreover, free choices that went against the distractors’ suggestion were associated with slower RTs than those that followed the suggestion (Fig 2A). This confirms that response conflict was triggered by distracting stimuli that were incongruent with participants’ choices, resulting in a cost to action selection. These findings are consistent with previous studies showing biases in free choices due to conscious [26] and unconscious [27–33] distracting stimuli.

In instructed trials, when participants had to follow the target’s direction, RTs were also slower if the distractors were incongruent with the target (and the required action) relative to congruent distractors (Fig 2A). Results further showed that RTs were slower in instructed than in free trials when the distractors were incongruent with the executed action, whereas RTs did not differ between free and instructed trials with congruent distractors. Therefore, the cost of conflict in instructed trials was larger than in free trials. This added difficulty may reflect the fact that instructed-incongruent trials involved both resolving conflict at the perceptual level, to correctly identify the target among the surrounding distractors (e.g. <<><<), as well as at a response level, between the competing responses triggered by the target and distractor stimuli. Such an interaction between choice and congruency has been previously reported with the flanker task [26]. Moreover, it could be argued that the visual stimuli might overall be more attended to in instructed, than free trials, rendering the distractors more salient. That is, participants might first assess whether the target indicated a free choice, allowing them to proceed with their value-based choice and ignore the stimuli, or an instructed trial, thus requiring further processing of the stimuli to categorise the target direction. Yet, we note that the absence of RT differences between free-congruent and instructed-congruent trials (Fig 2A), as well as between free and instructed-high value trials (Fig 3D), together with still observing effects of the distractors in free trials (Fig 2C), seem inconsistent with significant differences in attentional allocation to the stimuli across choice conditions (see also S4 Text). These results are rather more consistent with an online, adaptive allocation of cognitive control resources to handle conflict, whether due to low value instructions or to incongruent distractors, as and when deemed necessary.

Considering how actions were affected by recent conflict experience revealed the well documented conflict adaptation effect [14,15], of reduced conflict effects on RTs following conflict trials (Fig 2B). Although conflict effects were generally smaller in free trials (as discussed above), the reduction in the cost of conflict on RTs *following* conflict was similar in free and instructed trials. This is consistent with previous conflict triggering behavioural adjustments, such as greater attention to the middle target and/or suppression of distractors, leading to a similar reduction in the impact of conflicting distractors on RTs in the next trial, whether free or instructed. To the best of our knowledge, this is the first study to show such conflict adaptation effects in the context of intermixed free and instructed trials, and within a reinforcement learning task. These findings demonstrate the generalizability of such adaptation processes, which can be effective even in the presence of concurrent task demands (i.e. learning about action-reward contingencies).

In addition to benefiting from behavioural adjustments that could reduce subsequent conflict effects, free choice trials additionally offered an opportunity for making choices that avoided conflict altogether. Previous work has shown that both sequential conflict adjustments and avoidance can concurrently serve as behavioural strategies for minimising conflict experience [8,20]. The observed bias in participants’ choices to follow the distractors’ suggestions could thus be understood as resulting from a motivation to avoid conflict. This account is supported by the observation that choices were *more* biased by distractors following conflict trials (Fig 2C). This observation might have seemed to hint at a conflict cost to learning, since conflict avoidance was increased following conflict even when a positive outcome was obtained. However, the absence of robust effects of action-distractor conflict on learning in free trials speaks against this hypothesis (see S1 Text). Instead, we speculate this might result from recent conflict experience highlighting the aversive nature of conflict, thus increasing the motivation for avoiding conflict and cognitive demands. Interestingly, exploratory analyses on interindividual variability in behavioural adjustments following conflict (see S3 Text), showed that participants with a greater reduction in conflict effects on RTs also had a greater bias in choices. This supports the suggestion that both strategies can be used complementarily, to minimise conflict. Nonetheless, we note that these interpretations remain speculative, as sequential effects were not the primary focus of our study, but were rather assessed as potential “model-free” indexes for the dynamic decision and learning processes probed by our computational analyses.

It is worth emphasising that the probability of left and right distractors was equated, hence the distractors were equally likely to suggest the high or low reward action. Participants were made aware of that, and instructed to ignore the distractors. As we found that participants could learn to maximise their earnings, the distractor-related choice bias did not impair participants’ performance (see S3 Text for a further demonstrations that the distractor bias did not carry a relevant cost to performance). In fact, the observed learning curves (Fig 3A) suggest that the distractor bias was larger after reversals, when participants were more uncertain about which was the best action.

Our computational model (Fig 5), captured this effect by adding a "distractor bias" parameter, *φ*, to the decision rule (Fig 3E shows a clear correlation between the observed choice bias and our bias parameter). Although, theoretically, even small value differences could determine a decision, reinforcement learning models incorporate a degree of stochasticity in the decision rule through the temperature parameter, *β*, which is proportional to the value difference. In our model, the distractor bias parameter shifts the decision rule to increase the probability of distractor-congruent choices (if *φ* > 0, as observed), simultaneously reducing the probability of distractor-incongruent choices (Fig 3B). The distractor bias parameter could thus be interpreted as reflecting the subjective cost of conflict, as a larger bias value would require a larger expected reward to increase the probability of acting in conflict with the distractors. These findings are consistent with recent work demonstrating that the costs of exerting cognitive control are weighed against expected returns [43,72], similarly to other types of cognitive [3] and physical [6] effort discounting. Moreover, as stochasticity is greater for smaller value differences, our model naturally entails a greater influence of distractors on decisions with greater uncertainty about the best action (see Fig 3B).

It could be argued that the distractors could have *facilitated* the decision. As deciding between similarly valued options may be seen as a type of conflict in itself [32,50], the distractors’ suggestion could help break the tie between similar alternatives. The present results do not allow us to unequivocally arbitrate between the conflict avoidance vs. facilitation accounts. To avoid lengthening the experiment, the present study did not include trials with neutral distractors, that is, distractors that would not suggest either response option, e.g. using a double-headed arrow (currently the free choice target) also as flankers. Future studies with neutral distractors, and modelling differential facilitation and conflict bias parameters, could potentially dissociate an increased probability of distractor-congruent actions (facilitation) from a reduced probability of distractor-incongruent actions (conflict avoidance), relative to the neutral condition. Yet, these two accounts may not necessarily be incompatible.

From the perspective of decision-making as involving the accumulation of evidence for a response [73,74], both internal and external sources of information are integrated over time, until a decision bound is reached. As time-pressure was present in our study, delaying the response too much was counterproductive. Speed-accuracy trade-offs have been shown to influence evidence accumulation [75]. High expected values would lead to faster decisions than low values [41], and potentially to preparing the high value response *before* the trial. Such advanced preparation is supported by our findings that RTs were similar in free trials and when an instruction required making the subjectively best choice (Fig 3D), as both allowed participants to go ahead with the prepared action. In contrast, the presence of incongruent distractors would provide evidence for the alternative (low value) response, thus delaying the decision (i.e. slower RTs in incongruent than congruent trials, Fig 2A). When the initial evidence bias was high (given the expected value), recruiting control to suppress the effect of the distractors would be justified by enabling a quicker decision, since the accumulated evidence would remain closer to the decision bound of the high value option. Conversely, when the initial value difference was small, suppressing the distractors would return the accumulated evidence near the starting point, further delaying the response. In such cases, engaging cognitive control to suppress the distractors might carry the additional opportunity cost of foregoing any reward at all (as "too slow" responses constituted an error). The resulting facilitation of the decision by following the distractors’ suggestion would thus serve to avoid unnecessary/unjustified conflict.

Notably, accounts of the integration of information during the decision, whether emphasising facilitation effects or the avoidance of costs, may neglect processes happening before being presented with the decision context (i.e. before being presented with the choice alternatives & distractions). In fact, cost-benefit analyses could already guide the allocation of cognitive control in proactive manner [43], in order to *prevent* the biasing of free choices by the context. The motivation to avoid cognitive demands might be especially relevant at that point, as sufficiently high reward expectations could motivate sustaining proactive control. Such effects might be particularly relevant in our task, where the choice alternatives (left vs. right key), and hence the expected rewards, were known in advance (as noted above). Situations in which one must first inspect the available options to gage expected rewards might reduce the motivation to deploy proactive control. Finally, the perspective that cognitive control demands are perceived as aversive and costly helps understand their impact on behaviour more generally, not only on the allocation of cognitive control and decision-making, but also on outcome processing and learning.

Future studies employing a combination of drift-diffusion and reinforcement learning models [41], together with time-sensitive neuroimaging techniques (e.g. M/EEG), might yield important insights into the process of integrating multiple sources of information for decision-making, and of engaging cognitive control to deal with potential conflicts, both reactively and proactively. Such studies might thus help disentangle the subjective costs associated the specific cognitive control processes engaged in different situations, or time-points in a trial, such as detecting and resolving conflict between concurrent response activations, suppressing irrelevant or enhancing relevant information (stimulus- or value- based), or suppressing a pre-prepared response. Moreover, such measures could serve to index interindividual variability in conflict costs that could in turn account for variability in the impact on decision-making. As previously suggested [49], while some individuals might find cognitive demands to be aversive and thus seek to avoid cognitive effort, others might find cognitive demands to be invigorating and hence be motivated to invest more effort in the task.

### Influences on learning

Results showed that learning rates were higher in free choices than in instructed trials (Fig 3C). This suggests we might learn more about the consequences of actions that are driven by our own intentions and motivations. A greater sensitivity to rewards obtained through one’s choices over passively received rewards has been previously shown [76], and having a choice in what to do may itself be rewarding [77,78]. Moreover, an "illusion of control" has been demonstrated [77,79], wherein more favourable outcomes are expected for one’s choices, over when one has no choice. An increased value and reward expectation for one’s choices, combined with a tendency for learning more from positive feedback [80,81], may thus boost learning from free choices over instructed actions.

Research on learning and memory has shown improvements when people are allowed to decide how, or which items, to study, relative to not having a choice [82,83]. Being able to choose what to do, also referred to as self-directed learning, allows for more efficient deployment of resources to relevant information gathering [84], such as testing relevant hypotheses (i.e. is this really the best action?), in turn improving learning. When following instructions, one is exposed to information that may not seem particularly informative (if the other action yields a reward, that could just reflect a low probability outcome, rather than a reversal in contingencies). Furthermore, different neural mechanisms have been linked to learning from one’s choices relative to the choices of others [85,86], even when similar learning performance is demonstrated in a separate, post-learning, test. As our analyses focus on the dynamics of value updating in a frequently changing environment, they may emphasise differences in learning mechanisms from free vs. instructed actions.

Turning to the effects of conflict on reinforcement learning, our results suggest that the type of conflict experienced is important. Our task was primarily designed to induce conflict between one’s actions and external distracting information (i.e. flankers). Yet, in instructed trials, another type of conflict could be elicited between the instruction and subjective action values–i.e. what participants *had* to do vs. what they *wanted* to do. We found that both types of conflict disrupted action selection. Incongruent distractors led to slower RTs than congruent distractors, and triggered sequential conflict adaptation in RTs and choices (Fig 2). Conflict between instructions and subjective values also led to slower RTs, relative to when the instruction required the high value action or free choices (Fig 3D). Yet, whereas instruction-value conflict (in "instructed-low value" trials) led to a reduction in learning rates (Fig 3C), relative to no conflict ("instructed-high value" trials), we did not find robust evidence that action-distractor conflict modulated learning rates.

We suggest conflict between instructions and subjective values constitutes a type of motivational conflict. In those trials, participants were faced with two internal motivations competing to guide action selection: using subjective value information to make the best decision, vs. correctly following the instructions, to avoid losing potential rewards. We found that when participants had to suppress the drive to make what they perceived to be the best action, in order to correctly follow a subjectively "bad" instruction, they learned less from the observed outcomes. Thus, the conflict experienced during action selection seemed to devalue the action outcome. Such costs of motivational conflict to learning are consistent with previous studies involving conflict between Pavlovian tendencies and instrumental task requirements [64,65].

Despite the aforementioned commonalities in conflict monitoring across externally-triggered and motivational conflict [66,67], the absence of robust effects of action-distractor conflict on learning points to the relevance of remaining differences. For example, the differences in the specific cognitive control resources needed to resolve these two types of conflict might differ, and hence carry different subjective costs. Results showed that modelling different learning rates a function of action-distractor did not sufficiently improve model fit to justify the extra model complexity (see S1 Text for further consideration of the effects of this type of conflict on learning rates). It remains possible that the current design, or our sample size, limited our ability to detect an influence action-distractor conflict on learning. Additionally, the binary categorisation of trials into conflicted/non-conflicted (i.e. incongruent vs. congruent) may not have been sufficiently sensitive to trial-by-trial variations in the degree of conflict experienced. As mentioned above, future studies with more sensitive neural measures of conflict might help disentangle these accounts.

Alternatively, conflict triggered by irrelevant external stimuli may lead to more targeted conflict resolution mechanisms, focused on suppressing irrelevant information, in turn reducing conflict costs to outcome evaluation. Such conflict adaptation processes may be more difficult to engage, or less efficient, in the context of motivational conflicts. Whereas participants could systematically ignore the distractors in our task, they had to constantly switch between using subjective values to guide their decisions and following the task instructions (i.e. target direction). Therefore, in addition to the source of conflict, we speculate that the capacity for adaptation to conflict might be a relevant modulator of conflict costs to learning. Similarly, being able to choose *whether* to avoid conflict may also be relevant. Arguably, the observed absence of any effects of conflict on learning in free trials might provide some initial support for a moderating role of having a choice, and opportunity, to avoid conflict (see S1 Text for further consideration of this hypothesis, namely as an account for differences in findings relative to [50]).

### Conclusions

Our findings suggest that decision-making involves trade-offs between the expected value of a given course of action and the potential cognitive control costs incurred by that action. Unless there is a sufficiently good reason to handle it, e.g. expecting a reward, conflict is typically avoided. While experiencing conflict can sometimes influence subsequent processes, such as outcome evaluation, our results suggest that instrumental learning may not always be affected. We speculate that the effect of conflict on learning may be moderated by the type of conflict experienced, i.e. between competing internal drives or between internal vs. external information, as well as by the potential for conflict adaptation and avoidance.

## Materials and methods

### Ethics statement

The study was conducted in accordance with the declaration of Helsinki (1964, revised 2013), and was approved by a local ethics committee (CPP C07-28). Participants gave written informed consent before participating in the study.

### Participants

Twenty participants completed the study (10 females, mean age = 25.80 ±4.34). One participant had been recruited and completed the first session, but due to problems scheduling the second session, was excluded. Sample size was determined based on similar studies on RL [1,87], and ensured we had 80% power to detect a medium-sized, d = 0.6, choice bias effect induced by distractors (one-tailed, one sample case, at alpha = 0.05). We reasoned the effect here might be considerably smaller than a previously reported large choice bias effect (d = 1.31) in a similar flanker task but with arbitrary (i.e. not value-based) choices [26]. Notably, we collected a large number of trials (2 x 800 = 1600 trials) to maximise the within-subject sensitivity and reliability of our findings. Participants were told they would receive 15€ payment, and up to 5€ extra based on their performance, per session (~1.5 h). In fact, every participant received 20€ per session. All were right-handed, with normal or corrected-to-normal vision, did not suffer from colour blindness, and reported having no history of psychiatric or neurological disorders.

### Materials

Participants were seated approximately 50 cm from a computer screen. The experiment was programmed and stimuli delivered with Psychophysics Toolbox v3 [88–90], running on Matlab (MATLAB 8.1, The MathWorks Inc., Natick, MA, 2013). Stimuli were presented in black on a half grey background. A fixation dot was presented subtending 0.26° visual angle. Target and flanker stimuli consisted of left or right pointing arrows, subtending 0.6° visual angle, with a spacing of 0.1° between arrows. Participants responded by pressing one of two keys on a keyboard. Outcome stimuli consisted of "+1" or "-1", presented in 54 points Arial font. The error cross subtended 1° visual angle.

### Task

The reversal-learning task (Fig 1) required participants to continuously learn the reward probabilities (75%/25%) associated with left vs. right hand actions, and adapt their choices accordingly. For example, right hand actions might have a 75% probability of yielding a reward (+1 point), whereas left hand actions would yield a reward in only 25% of trials. The remaining trials were associated with a loss (-1 point). This task was combined with a flanker task, such that participants responded to a target arrow, which appeared surrounded by irrelevant distractor (i.e. flankers). Participants were explicitly instructed to ignore these distractors, and focus on the middle arrow. Each trial started with a 400 ms fixation dot, followed by a 100 ms blank screen. The target and distractor array was displayed until a response was made, or up to 1.2 s. In free trials, the middle arrow consisted of two overlapping left/right pointing arrows, indicating that participants were free to choose which action to make. Trials were classed as *congruent* if participants chose the action that corresponded to the direction of the distractors, and as *incongruent* if participants chose the opposite action to the distractors. In instructed trials, the target arrow consisted of a left or right pointing arrow, and participants had to respond according to its direction. Distractors could be congruent or incongruent with the target direction. If participants responded correctly, after a brief interval of 300 ms, the reward outcome (+1/-1) was displayed for 700 ms. The inter-trial interval varied randomly between 0.8–1.2 s. If participants made the wrong action in instructed trials, or did not respond within 1.2 s, an error cross was immediately displayed for 700 ms.

After an unpredictable number of trials, the mapping of action to reward probabilities was reversed. After a reversal, the best-rewarded response (e.g. right) became the least-rewarded response, and vice-versa. The length of reversal episodes, i.e. number of trials before a reversal, followed a pseudo-gaussian distribution ([8, 8, 16, 16, 16, 24, 24, 24, 32, 32]). To ensure that all conditions (free vs. instructed, congruent vs. incongruent) were adequately counterbalanced within each episode, the same number of free and instructed trials was included. In instructed trials, we ensured equal numbers of left/right congruent/incongruent trials. As we could not control congruency in free trials, we presented equal numbers of left and right pointing distractor. Outcome probability was equated across congruency conditions for the instructed trials. Across the experiment, we counterbalanced the condition of the first trial in an episode (free/instructed, left/right distractors, and left/right instructed actions). The distribution of episode lengths and type of trial at the start of the episode was pseudo-randomised, such that all combinations of lengths and types were randomised before being repeated. We additionally ensured that the same type/length (or trial type in randomising trials) was not repeated more than 3 times in a row. Participants completed a total of 1600 trials, across 2 sessions (separated by ~5 days, range: 1–8). Breaks were introduced approximately every 15 mins, for 10 s. Participants were instructed that the breaks were independent of changes in reward probabilities.

Participants were instructed that they would play a game in which they could try to earn as many points as possible by making the best choice. They were told that the points they accumulated could allow them to earn a bonus (5€) at the end of the study, as a function of their performance, and relative to a target level of points. More specifically, the participants were told that the goal of the game was to discover which key (left or right) would enable them to maximise their financial earnings, as one of the keys would, on average, be more advantageous than the other, and would yield gains (+1) more often. Participants were told that the best key (i.e. most rewarding) was the same when choosing freely or following instructions. Therefore, they should pay attention to the outcomes of instructed choices, as that gave them information about which was currently the best key. They were cautioned that, at various time points and without notice, the best key would become the worst key (least rewarding), and that they should adapt to these changes. To ensure participants responded correctly in instructed trials, they were informed that errors–pressing the wrong key in instructed trials, or too late–would reduce their chances of winning the final bonus, and hence they should try their best to minimise the number of errors made. The instructions about how point earnings and errors would be translated into monetary earnings remained vague, as the “target level” for the bonus remained undisclosed, and participants did not receive any feedback about their point earnings, nor error frequency, during the task. This served to prevent participants from strategically trying to compute whether making an error in instructed trials might be more beneficial, especially when having to follow a subjectively “bad” instruction (i.e. “instructed-low value” trials).

Before the main experiment, participants completed some training blocks. During training, the reward probabilities were made more easily distinguishable (87.5% vs. 12.5%). Additionally, in the first training block, the best action was cued by displaying the corresponding target arrow in green (when the option was available). This served to help participants understand the probabilistic nature of the rewards, and track the reversal of the reward probabilities. In the second training block, the best action was no longer cued, thus all arrow stimuli were presented in black, as in the main experiment.

### Behavioural analyses

Trials without a response within 1.2 s were excluded (free: 0.90% ±2.08; instructed: 0.71% ±1.83). In instructed trials, the percentage of error trials was analysed as a function of distractor-target congruency, and errors were excluded from further analyses. Other statistical tests are described in the results section.

### Computational models

We fitted the data with a standard *Q*-learning model [69,70]. The model estimates the expected values (*Q*-values) of the two possible actions (left vs. right hand). The *Q*-values were set to 0 before each learning session, corresponding to the *a priori* expectation of a 50% chance of winning 1 point, plus a 50% chance of losing 1 point. After each trial *t*, the value of chosen option was updated according to the following rule:

Q(t+1)=Q(t)+α*(R(t)-Q(t))

where *R*(*t*) was the reward obtained for the chosen option at trial *t*, and *α* referred to the learning rate parameter.

A softmax rule was adapted to estimate the probability of selecting a particular option (e.g. right) as a sigmoid function of the difference between the net values of left and right options, with a temperature parameter *β*, which captures choice stochasticity. To capture the influence of the distractors at the decision-making stage, we added a free parameter *φ*, which biased the choice depending on the relation between the distractors and that option at trial *t* as follows:

$$P_{Right}(t)=\frac{1}{1+e^{\left(\beta *(Q_{Left}(t)-Q_{Right}(t))-\varphi *C(t)\right)}}$$

where *C*(*t*) = 1 if distractors were congruent with that option (i.e. point to the right), and *C*(*t*) = −1 if distractors were incongruent with the option. Thus, the estimated parameter *φ* > 1 indicates that distractors biased choice toward the congruent option; *φ* < 1 indicates that distractors biased choice toward the incongruent option; or *φ* = 0 indicates that the distractors had no influence on choice.

As the model-free analyses revealed that free choices were significantly biased by the distractors, we considered it critical to capture this influence at the decision stage in our models. Therefore, apart from considering the simplest, standard reinforcement learning model (with only two free parameters [*β*, *α*]), the remaining models in our model space included the *φ* parameter in the decision rule (see S2 Text for evidence that this parameter is needed to adequately capture participants’ behaviour). To assess the potential influence of the choice and congruency manipulations on learning, we considered models that varied in the number of learning rates, from a single learning rate, to different learning rates as a function of choice, congruency, and their interaction.

In an extra model (m8), we estimated separate learning rates as a function of choice, and as function of subjective beliefs for instructed trials. That is, we used the estimated difference in action values (Δ*Q = Q*_*L*_*−Q*_*R*_) to split trials with instructions to make the (subjectively) high vs. low value action. For example, if participants followed an instruction to go right:

$$Q_{R}(t+1)=\left\{ \begin{array}{c} Q_{R}(t)+\alpha_{Instructed\_High\;Value\;}*(R(t)–(Q_{R}(t))\;\text{if}\;\Delta Q>0\\ {\;Q}_{R}(t)+\alpha_{Instructed\_Low\;Value}*(R(t)–(Q_{R}(t))\;\text{else} \end{array}\right.$$

### Parameter optimisation and model comparison

Model parameters were optimised by minimising the negative log-likelihood of the data, given the parameters settings (using Matlab fmincon function, ranges: 0 < *β* < +Infinite, -Infinite < *φ* < +Infinite, and 0 < *α*_*n*_ < 1). To compare models fits while accounting for the model complexity of adding extra free parameters, we calculated Aikake Information Criteria (AIC) based on the negative log-likelihoods for each participant, and each model, as follows:

AIC=-2*LogLikelihood+2*df

where *df* refers to the number of free parameters. In this study we opted for the AIC over the BIC, because the latter criterion tended to over-penalized complex models.

AIC values were then used as an approximation to the log model evidence [91], and models were treated as a random variable in a group-level variational Bayes analysis for model selection (using the "VBA toolbox" [92]). This approach allows for the estimation of the expected model frequency, and exceedance probability of each model, within the model space and given the data from all participants [93]. Expected model frequency quantifies the posterior probability of the model, i.e. the likelihood that the model generated the data of a random subject in the population. The exceedance probability (*xp*) quantifies the probability that a given model fits the data better than all other models in the set, i.e. has the highest expected frequency.

Following previous work [87,94], we conducted an additional optimisation procedure that minimised the logarithm of the Laplace approximation to the model evidence, often referred to as maximum a posteriori or log posterior probability (LPP). This approach avoids degenerate parameters estimates as it includes priors over the parameters (Gamma(1.2,5) for *β*; Normal(0,1) for *φ*; and Beta(1.1,1.1) for *α*_*n*_). Further analyses (and figures) are based on the parameters estimated though this procedure.

### Validation of the model and parameter optimisation

To ensure that our task design and parameter optimisation procedure would indeed be able to identify potentially dissociable effects of distractor-action conflict, as hypothesised, we used simulated virtual data based on pre-defined parameter values that should be recovered by the running the optimisation procedure on the simulated data. If our design or optimisation procedure were flawed, and would mistakenly introduce biases in the estimated parameters, then the recovered parameter values would differ from the parameters used in the "virtual participants". We simulated virtual datasets (*N* = 100) based on five sets of parameter values (while holding a constant *β* = 2). These aimed to capture three hypothetical results of action-distractor conflict: A) a cost of conflict on decision [*φ* = 0.2, *α*_*C*_ = *α*_*I*_ = 0.6], but no effect on learning; B) a cost of conflict on learning [*φ* = 0, *α*_*C*_ = 0.7, *α*_*I*_ = 0.5]; C) a benefit of conflict to learning [*φ* = 0, *α*_*C*_ = 0.5, *α*_*I*_ = 0.7]. We additionally tested parameter sets simulating effects on the decision and on learning simultaneously (D: [*φ* = 0.2, *α*_*C*_ = 0.7, *α*_*I*_ = 0.5]; E: [*φ* = 0.2, *α*_*C*_ = 0.5, *α*_*I*_ = 0.7]) These datasets were then submitted to the parameter optimisation procedure for the simplest model that could assess an effect of conflict on the decision and on learning (i.e. m3 with the free parameters [*β*, *φ*, *α*_*C*_, *α*_*I*_]). The estimated parameters are displayed in Fig 4. These results clearly show that the simulated parameter values were adequately recovered. Moreover, it shows that effects at the decision and learning stages are, at least theoretically, dissociable (whether simulated separately or simultaneously). Consequently, the effects observed on our real participants (i.e. a cost of action-distractor conflict on the decision, but no effect on learning rates) cannot be attributed to our design or parameter estimation procedure introducing specific biases for or against finding particular effects.

## Supporting information

S1 Text Effect of action-distractor conflict on learning. (PDF)

S2 Text The critical role of the distractor bias parameter. (PDF)

S3 Text Distractor bias parameter vs. behaviour correlations. (PDF)

S4 Text Effect of distractor vs. value conflicts in action selection in instructed trials. (PDF)

S1 Table Reduced model space comparison results. β refers to choice temperature parameter, reflecting choice stochasticity. φ refers to the distractor bias parameter added to the decision rule. α refers to the learning rate, split by conditions. Winning model (m4) highlighted in bold. RL = Reinforcement Learning, C = Congruent, I = Incongruent. AIC = Akaike Information Criteria, SD = Standard Deviation.
(PDF)
