# Supplementary material for: Cost-benefit trade-offs in decision-making and learning
Source: PLoS Comput Biol. 2019 Sep 6;15(9):e1007326. doi: 10.1371/journal.pcbi.1007326 (PMC6750595; doi:10.1371/journal.pcbi.1007326)
Supplement: S1 Text — (PDF) [file pcbi.1007326.s001.pdf]

## S1 Text. Effect of action-distractor conflict on learning

For the sake of full disclosure and transparency, we present here a summary of the estimated parameters obtained by m7 – a model with separate learning rates as function of the interaction between choice and distractor-action congruency, plus the distractor bias parameter in the decision rule. Since this model did not win over the others in the models comparison, due to the extra model complexity, these results are only suggestive, and should be interpreted with care. We still thought it important to share these results, since the only weak evidence we found for an effect on action vs. distractor conflict on learning would be a benefit to learning. This goes against the hypothesis that conflict generally carries a cost to learning, due to its aversive nature, and is the opposite of what we found for conflict between instructions and subjective values. Finally, although the hypotheses discussed here remain speculative, they may offer relevant ideas for future research.

As in the other models, the estimated distractor bias parameter was significantly different from 0 (average $\varphi$ = 0.17 ± 0.25, $t_{19}$ = 3.05, p = .007, d = 0.96). The estimated learning rates (**Fig A**) were submitted to a repeated-measures ANOVA (choice: free vs. instructed; distractor-action congruency: congruent vs. incongruent). This showed a significant main effect of choice ($F_{1,19}$ = 15.56, $p$ < .001, $\eta_p^2$ = 0.45), as learning rates were lower in instructed than free choices. It additionally showed a significant main effect of distractor-action congruency ($F_{1,19}$ = 14.98, $p$ = .001, $\eta_p^2$ = 0.44), with higher learning rates in incongruent than congruent trials. Yet, these main effects were qualified by a significant choice-by-congruency interaction ($F_{1,19}$ = 5.94, $p$ = .02, $\eta_p^2$ = 0.24). Post-hoc tests revealed that learning rates were significantly higher in incongruent than congruent trials in instructed trials ($t_{19}$ = -4.85, $p$ < .001, $d$ = -1.08), but there was no significant effect of congruency in free trials ($t_{19}$ = -0.12, $p$ = .906, $d$ = -0.03). Moreover, learning rates were always lower in instructed than in free trials (free-congruent vs. instructed-congruent: $t_{19}$ = 4.28, $p$ < .001, $d$ = 0.96; free-incongruent vs. instructed-incongruent: $t_{19}$ = 3.14, $p$ = .005, $d$ = 0.70).

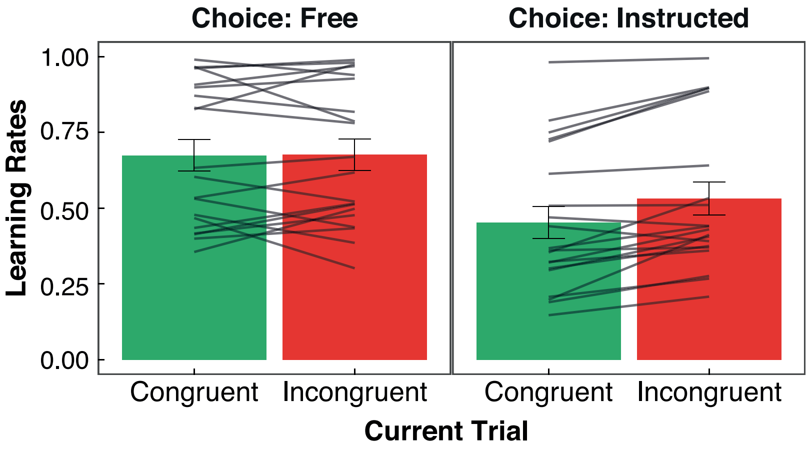


**Fig A. Estimated learning rates in m7.** Average learning rates ($\alpha$) in m7 as a function of choice and current trial distractor-action congruency. Error bars represent the standard error of the mean, and the lines represent each participant.

These results suggest that in instructed trials, the conflict triggered by incongruent distractors and targets might have led to an increase in learning rates, relative to no conflict. Yet, in free trials, action vs. distractor conflict did not influence learning. Recall that we found larger RTs cost in instructed than free trials due to conflict (see Results section). We hypothesised this was related to increased conflict costs associated with resolving both perceptual conflict (target vs. distractors), and at a response level (simultaneous activation of both responses), whereas in free choices, there was only conflict at the response level. This combined perceptual and response conflict in instructed trials may have required the deployment of more attentional resources to focus on the relevant stimuli, than in free trials. This might in turn result in enhanced attention at the time of the outcome, and thus in higher learning rates in incongruent than congruent trials.

It could have been argued that the absence of conflict effects on learning rates in free relative to instructed trials would be linked to differences in the effect sizes. RTs were slower in incongruent than congruent trials by an average of 66 ms in instructed trials, but only around 28 ms in free trials. This reduced effect might have thus been too weak to influence learning. However, the effects we found on learning rates due to conflict between instructions and subjective values (in m8) were associated with around 25 ms conflict costs on RTs (instructed low *minus* high value), similar to the cost of distractor-action conflict in free trials. Hence, the relatively smaller RTs costs in free trials cannot explain the absence of an effect on learning.

Therefore, the absence of effects of conflict on learning in free trials might rather be due to conflict being dealt with differently. Although free choices were still disrupted by incongruent distractors (evidenced by slower RTs, **Fig 2A**), such choices were likely driven by large differences in action value (as implied by our model, **Fig 3B**). We speculate that such chosen conflict might be subjectively experienced as different from imposed (or unavoidable) conflict. The extra effort might seem "justified" by the expected action values, rendering it less aversive, and cancelling out potential conflict costs on learning [1]. Furthermore, as participants might generally devote more attention to the task in free trials, due to a greater perceived relevance of information (as seen in the choice effect on learning rates), the attention at the time of the outcome might not be further modulated. In contrast, if participants are less engaged in the task in instructed trials, but are then obliged to pay attention to the task to successfully resolve conflict, this enhanced attention may then improve outcome processing, relative to the instructed-congruent trials.

We further suggest that having a choice in whether to experience conflict may partially explain why we did not find conflict costs on obtained rewards as previously reported with the Simon task [2], which also involves externally-triggered conflict. As mentioned in the introduction, during the learning phase of that study [2], participants had to respond according to stimuli, some of which were associated with conflict. Thus, the fact that conflict was unavoidable might have increased its subjective cost. Furthermore,

their design closely mirrored other effort discounting tasks, wherein people learn how much effort is needed to obtain a reward and, subsequently, show a preference for low effort options [3]. In contrast, in our study, conflict with distractors was fully orthogonal to the learning task. This allowed us to investigate how learning might be dynamically influenced by an unpredictable, and task-irrelevant, experience of conflict, rather than offering the opportunity to learn to predict upcoming conflict. Future work is clearly needed to investigate the conditions under which conflict might discount rewards, or be its effects may be cancelled out by other mechanisms.

## References


1. Schouppe N, Braem S, Houwer JD, Silvetti M, Verguts T, Ridderinkhof KR, et al. No pain, no gain: the affective valence of congruency conditions changes following a successful response. Cogn Affect Behav Neurosci. 2015;15: 251–261. doi:10.3758/s13415-014-0318-3

2. Cavanagh JF, Masters SE, Bath K, Frank MJ. Conflict acts as an implicit cost in reinforcement learning. Nat Commun. 2014;5: ncomms6394. doi:10.1038/ncomms6394

3. Vassena E, Silvetti M, Boehler CN, Achten E, Fias W, Verguts T. Overlapping Neural Systems Represent Cognitive Effort and Reward Anticipation. PLoS ONE. 2014;9: e91008. doi:10.1371/journal.pone.0091008
