# Supplementary material for: Cost-benefit trade-offs in decision-making and learning
Source: PLoS Comput Biol. 2019 Sep 6;15(9):e1007326. doi: 10.1371/journal.pcbi.1007326 (PMC6750595; doi:10.1371/journal.pcbi.1007326)
Supplement: S2 Text — (PDF) [file pcbi.1007326.s002.pdf]

## S2 Text. The critical role of the distractor bias parameter

To further test the need to consider an effect of distractors at the decision stage, we assessed whether an alternative model without that parameter was able to reproduce the behaviour observed in our participants. For this, we compared the generative performance of the winning model (m8) to an alternative model (m8_alt), which differed only in that the decision rule followed the standard softmax rule (i.e. without the added $\varphi$, equivalent to $\varphi = 0$; but with the remaining free parameters as in m8: [$\beta$, $\alpha_{Free} \neq \alpha_{Instructed\_High\ Value} \neq \alpha_{Instructed\_Low\ Value}$]). First, we fitted this model to the real data. Next, we used the average estimated parameter values to simulate data ($N$ = 100). **Fig B** shows the simulated data from the alternative model (m8_alt), overlaid over the real data and simulations from the winning model (m8, already displayed in **Fig 3A**). This shows that the alternative model, without the distractor bias parameter, fails to qualitatively reproduce the observed distractor bias effect, i.e. higher proportion of distractor congruent than incongruent choices, from the start of learning episodes. This bias is instead clearly observed in simulations of the winning model (m8), which included the distractor bias parameter.

Therefore, we believe the poor generative performance of the alternative model, which fails to capture an important behavioural effect, is sufficient cause for rejecting it as a suitable model [1], hence excluding other models without a distractor bias from our model space.

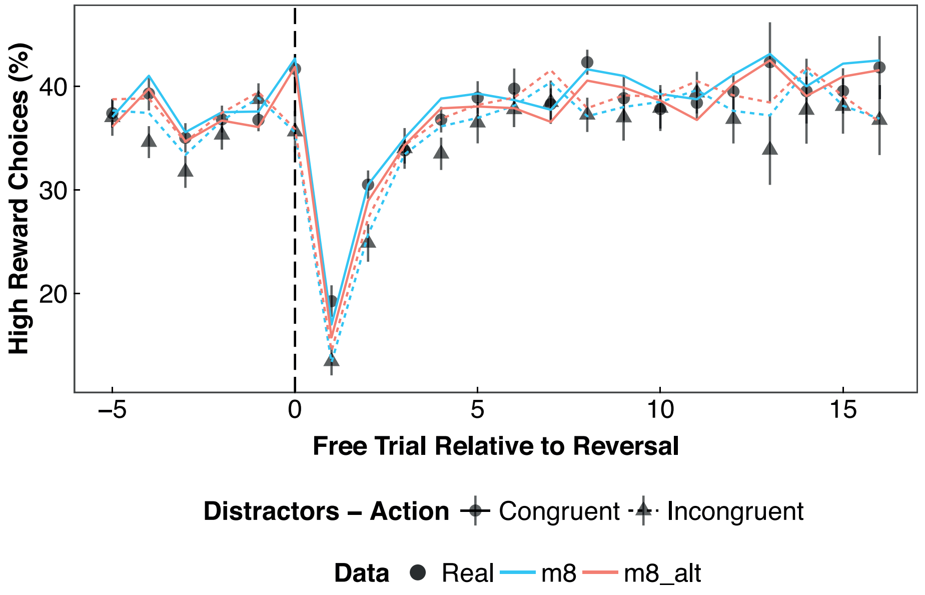


**Fig B. Model simulations.** Simulations for the winning model (m8) and a similar model without the distractor bias ($\varphi$) parameter as an alternative model (m8_alt). Dots and error bars represent real participants' data, while the lines represent the average of each model simulation.

### References

1. Palminteri S, Wyart V, Koechlin E. The Importance of Falsification in Computational Cognitive Modeling. Trends Cogn Sci. 2017; doi:10.1016/j.tics.2017.03.011
