# Supplementary material for: Cost-benefit trade-offs in decision-making and learning
Source: PLoS Comput Biol. 2019 Sep 6;15(9):e1007326. doi: 10.1371/journal.pcbi.1007326 (PMC6750595; doi:10.1371/journal.pcbi.1007326)
Supplement: S3 Text — (PDF) [file pcbi.1007326.s003.pdf]

S3 Text. Distractor bias parameter vs. behaviour correlations

To investigate the relation between conflict avoidance and conflict adaptation effects, we assessed the relation between the estimated distractor bias parameter (as an index conflict avoidance) and conflict adaptation on RTs. Conflict adaptation effects were calculated as the difference between conflict effects (I *minus* C) for previously *congruent* minus previously incongruent trials. Thus, larger conflict adaptation reflects a greater reduction in conflict effects following incongruent trials. Since similar conflict adaptation was observed for free and instructed trials, we averaged over choice conditions. This analysis revealed a significant positive correlation between the distractor bias parameter, i.e. conflict avoidance, and conflict adaptation effects on RTs (see **Fig C.i**, Pearson's correlation: r = 0.54, t_{18} = 2.72, p = .014). That is, participants who were better able to adapt their behaviour to reduce conflict costs on RTs were also more likely to avoid conflict when unnecessary (i.e. in the absence of strong value differences).

These results should be interpreted with care, given our relatively small sample size. Nonetheless, they suggest that participants' sensitivity to conflict may be reflected in these two types of adaptive behaviours, rather than being a trade-off between them. It could have been hypothesised instead that participants who were worse at minimising RT costs would benefit most from avoiding conflict. Yet, this correlation implies that a common process of conflict monitoring and adaptation may underlie both types of behavioural responses. In fact, previous work has suggested that conflict signals can trigger both adjustments in cognitive control and conflict avoidance ([1–3] but see [4]).

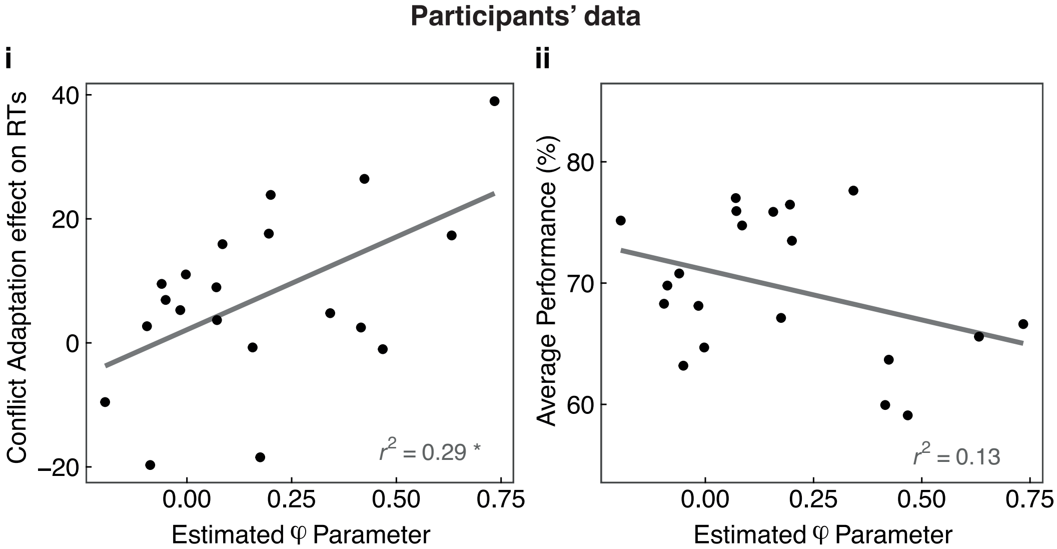

Fig C. Relation between distractor bias parameter and participants' behaviour. Correlations between the estimated distractor bias parameter (φ) and conflict adaptations effects on RTs (**i**), and average performance (**ii**).

Finally, it could have been hypothesised that having a larger choice bias might impair performance in the task, as participants choices might be too driven by the distractors

rather than action values. Importantly, since the probably of left and right distractors was equate within each learning episode (i.e. between reversals), following the distractors' suggestion would be equally likely to be helpful vs. unhelpful to task performance (i.e. 50/50 chance). Nevertheless, we tested this hypothesis by assessing the correlation between the estimated distractor bias parameter and average task performance, which showed no significant correlation (**Fig C.ii**, Pearson's correlation: r = -0.36, t_{18} = -1.63, p = .12).

The independence of distractor bias effects from average performance was further corroborated through model simulations. Virtual datasets (N = 100) were simulated across a range of distractor bias (φ) values ([-2, 2], at intervals of 0.1; and constant β = 2, α = 0.6). The simulated virtual choices were then used to calculate the average performance (i.e. average proportion of high reward choices, **Fig D.i**), as well as the percentage of distractor congruent choices (**Fig D.ii**), i.e. the consequence of the simulated distractor bias effect. These findings show that, across this broad range of φ values (φ estimated on participants' data varied less than 1 unit), changes in average performance were minimal, whereas they were associated with very large differences in the effect of distractor bias on free choices (i.e. proportion of distractor congruent vs. incongruent choices). This confirms that neither our task nor our model implies that the distractor bias would result in a significant impairment in task performance.

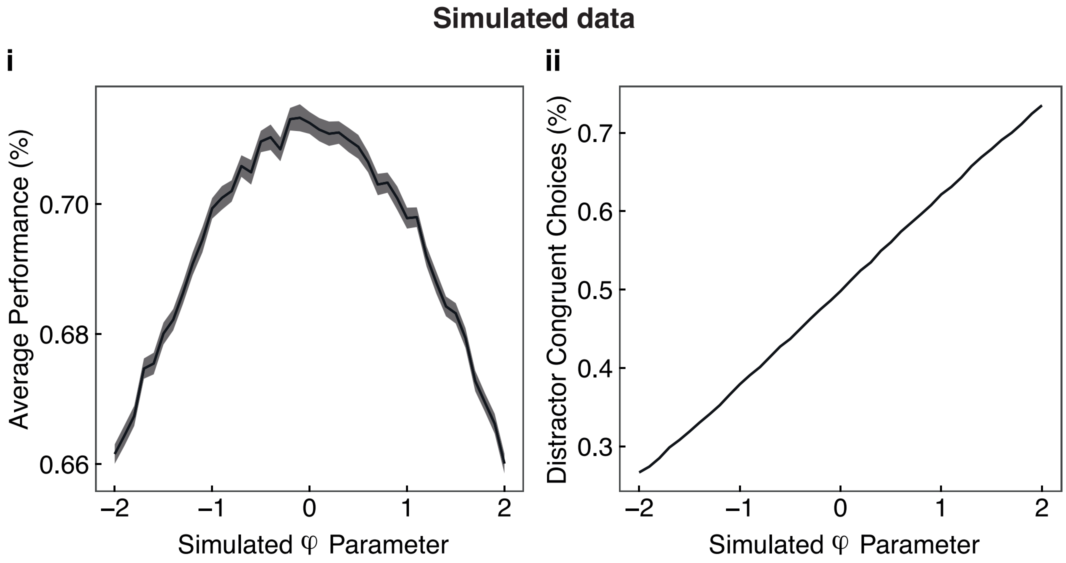

Fig D. Effect of varying distractor bias parameter on simulated behaviour.
Simulated data to assess the effect of varying the distractor bias parameter (range of [-2, 2], at intervals of 0.1; constant β = 2, α = 0.6) on average performance (**i**), and on the percentage of distractor congruent choices (**ii**).

References

1. Botvinick MM. Conflict monitoring and decision making: Reconciling two perspectives on anterior cingulate function. Cogn Affect Behav Neurosci. 2007;7: 356–366. doi:10.3758/CABN.7.4.356

2. Dignath D, Kiesel A, Eder AB. Flexible conflict management: Conflict avoidance and conflict adjustment in reactive cognitive control. J Exp Psychol Learn Mem Cogn. 2015;41: 975–988. doi:10.1037/xlm0000089

3. Dreisbach G, Fischer R. If it's hard to read… try harder! Processing fluency as signal for effort adjustments. Psychol Res. 2011;75: 376–383. doi:10.1007/s00426-010-0319-y

4. Schouppe N, Ridderinkhof KR, Verguts T, Notebaert W. Context-specific control and context selection in conflict tasks. Acta Psychol (Amst). 2014;146: 63–66. doi:10.1016/j.actpsy.2013.11.010
