# Supplementary material for: Cost-benefit trade-offs in decision-making and learning
Source: PLoS Comput Biol. 2019 Sep 6;15(9):e1007326. doi: 10.1371/journal.pcbi.1007326 (PMC6750595; doi:10.1371/journal.pcbi.1007326)
Supplement: S4 Text — (PDF) [file pcbi.1007326.s004.pdf]

S4 Text. Effect of distractor vs. value conflicts in action selection in instructed trials

In instructed trials, participants could experience both (a) action-distractor conflict, and (b) instruction-value conflict. Although participants were instructed to avoid making errors, as these would reduce their final earnings, participants could have still thought that the errors might be less costly than losing 1 point (i.e. seeing the "-1" feedback, rather than the error "X"). Consequently, it remained possible that they might strategically choose to commit errors in instruction-value conflict trials (i.e. instructed-low value), to avoid the loss feedback.

To investigate these hypotheses, we compare the influence of two types of conflict in action selection in instructed trials, submitting reaction times (RTs) and error rates to a 2 x 2 repeated-measures ANOVA, as a function of current trial distractor-action congruency: congruent vs. incongruent, and of the expected value of the instructed action: high vs. low. As for the RT analysis shown in **Fig 3D**, trials were split into high vs. low value instructions based on simulating action values (Q values) in each trial, for each participant (based on each participant's estimated parameters of m8).

Reaction Times

Analysis of RTs showed a significant main effect of congruency ($F_{1,19} = 145.95$, $p < .001$, $\eta_p^2 = 0.88$), a significant main effect of action value ($F_{1,19} = 28.65$, $p < .001$, $\eta_p^2 = 0.60$), and a significant interaction effect ($F_{1,19} = 8.00$, $p = .01$, $\eta_p^2 = 0.30$). This shows that both factors influenced RTs. Moreover, as seen in **Fig E.i**, congruency had a larger effect on RTs (~66ms slower in incongruent vs. congruent trials), overall, than instructed action values (~25ms slower in low vs. high value trials). Post-hoc tests revealed that all pairwise comparisons were significantly different (all $ps < .001$). The interaction effect revealed that the congruency effect (I-C) was significantly larger in high (~71ms) than in low (~61ms) value trials ($t_{19} = 2.83$, $p = .011$, $d = 0.63$).

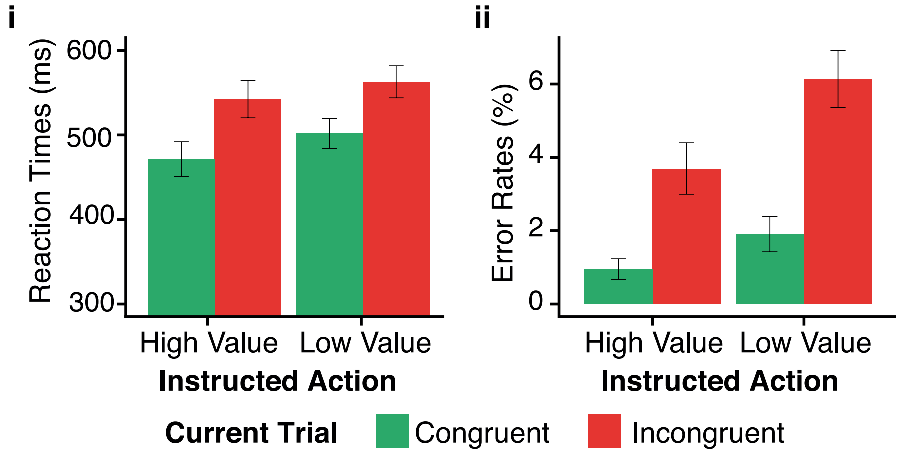

Fig E. Action selection in instructed trials. Average reaction times (**i**) and error rates (**ii**) in instructed trials as a function of distractor-action and instruction-value conflicts. Error bars represent the standard error of the mean.

Error Rates

Analysis of the error rates showed a significant main effect of congruency ($F_{1,19}$ = 33.19, p < .001, η_p^2 = 0.64), a significant main effect of action value ($F_{1,19}$ = 16.02, p < .001, η_p^2 = 0.46), and a marginal interaction effect ($F_{1,19}$ = 3.66, p = .07, η_p^2 = 0.16). This shows that participants made more errors in incongruent trials, as well as in trials in which they were instructed to make the subjectively low value action. As seen in **Fig E.ii** congruency had a larger effect on errors (~3.5% more errors in incongruent trials), overall, than instructed action values (~1.7% more errors in low value trials).

As the interaction effect remained marginal, and we did not have specific *a priori* hypotheses about it, it was not tested further. Inspection of **Fig E.ii** points to a relatively larger congruency effect for low (~4.2%) than for high value (~2.7%) instructed actions. This could suggest that it was especially hard for participants to respond correctly when they experienced combined conflict between target and distractors, and between target instruction and internal values. This scenario (incongruent-low value) involves both the salient distractors and the internal values activating the same action (e.g. right), thus potentially requiring more cognitive control to suppress the inadequate activation in order to respond correctly (e.g. left), though this interpretation remains speculative.

Summary

In short, incongruent distractors were the predominant cause for disruption of action selection, as evidenced by increased error commission and slowing down RTs. This is consistent with participants generally trying to follow the instructions, but occasionally being too impulsive to suppress their internal motivations, rather than participants strategically committing errors when they disagreed with the instruction.
