# Supplementary material for: Cost-benefit trade-offs in decision-making and learning
Source: PLoS Comput Biol. 2019 Sep 6;15(9):e1007326. doi: 10.1371/journal.pcbi.1007326 (PMC6750595; doi:10.1371/journal.pcbi.1007326)
Supplement: S1 Table — β refers to choice temperature parameter, reflecting choice stochasticity. φ refers to the distractor bias parameter added to the decision rule. α refers to the learning rate, split by conditions. Winning model (m4) highlighted in bold. RL = Reinforcement Learning, C = Congruent, I = Incongruent. AIC = Akaike Information Criteria, SD = Standard Deviation. (PDF) [file pcbi.1007326.s005.pdf]

S1 Table. Reduced model space comparison results.

**Models**	**AIC** ± SD	**Model Frequency**	**Exceedance Probability (*xp*)**
m1: Standard RL [β, α]	723.3 ± 191.4	0.14	0.02
m2: [β, φ, α]	714.3 ± 183.5	0.10	0.00
m3: [β, φ, $\alpha_C \neq \alpha_I$]	715.2 ± 184.0	0.06	0.00
**m4: [$\boldsymbol{\beta}$, $\boldsymbol{\varphi}$, $\boldsymbol{\alpha_{Free} \neq \alpha_{Instructed}}$]**	**704.7 ± 176.8**	**0.43**	**0.97**
m5: [β, φ, $\alpha_{Free\_C} \neq \alpha_{Free\_I} \neq \alpha_{Instructed}$]	704.9 ± 178.4	0.09	0.00
m6: [β, φ, $\alpha_{Free} \neq \alpha_{Instructed\_C} \neq \alpha_{Instructed\_I}$]	705.2 ±177.4	0.06	0.00
m7: [β, φ, $\alpha_{Free\_C} \neq \alpha_{Free\_I} \neq \alpha_{Instructed\_C} \neq \alpha_{Instructed\_I}$]	705.4 ± 178.8	0.12	0.01

β refers to choice temperature parameter, reflecting choice stochasticity. φ refers to the distractor bias parameter added to the decision rule. α refers to the learning rate, split by conditions. Winning model (m4) highlighted in bold. RL = Reinforcement Learning, C = Congruent, I = Incongruent. AIC = Akaike Information Criteria, SD = Standard Deviation.
